# Supplementary figures and images for: Engineered Nanobodies for early and accurate diagnosis of dengue virus infection
Source: PLoS Negl Trop Dis. 2025 Oct 31;19(10):e0013168. doi: 10.1371/journal.pntd.0013168 (PMC12591456; doi:10.1371/journal.pntd.0013168)

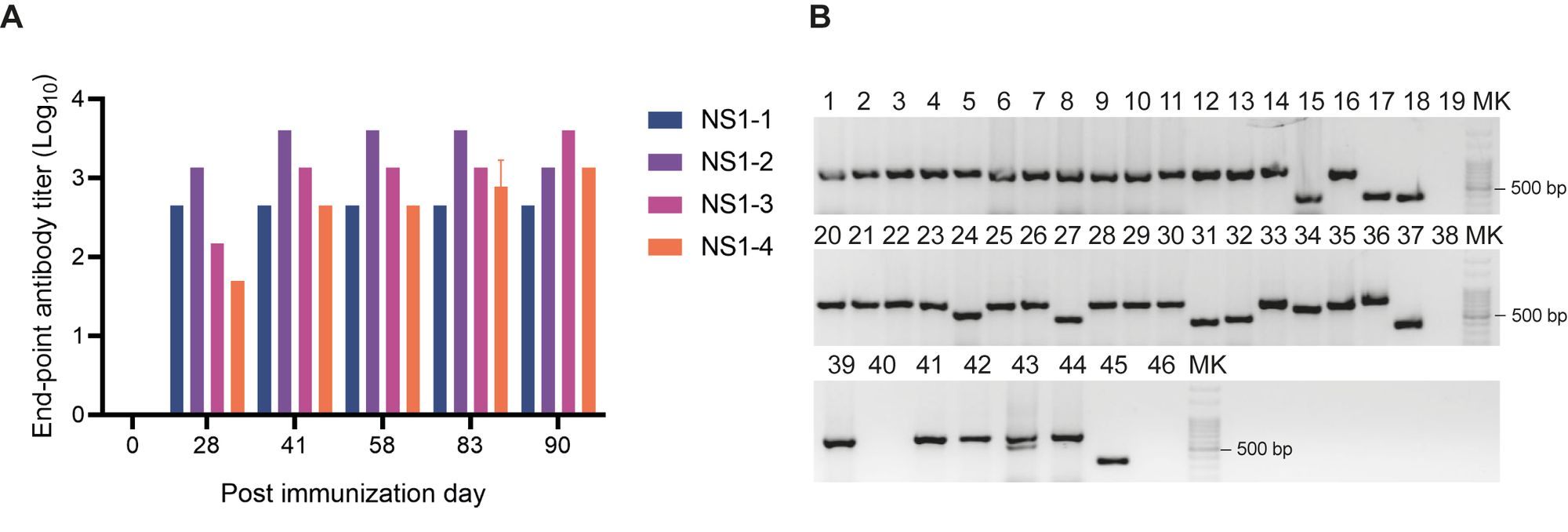

Supplement: S1 Fig — A-IgG antibody titers were evaluated before and after immunization with purified supernatants containing the NS1 protein from each DENV strain. Higher titers were achieved for NS1-2 (dilution 1/1350), NS1-3 (dilution 1/4050) and NS1-4 (dilution 1/1350). In contrast, the anti-NS1-1 titer showed no significant increase even after multiple immunizations (dilution 1/450). B-Agarose gel displaying ~700 bp PCR fragments corresponding to VHHs of varying sizes, and ~300 bp PCR fragments corresponding to empty vectors amplified from randomly selected individual colonies. The efficiency of the library was determined to be 78.5% (33 out of 42 colonies contain fragments incorporating a VHH sequence). (JPG) [file pntd.0013168.s004.jpg]

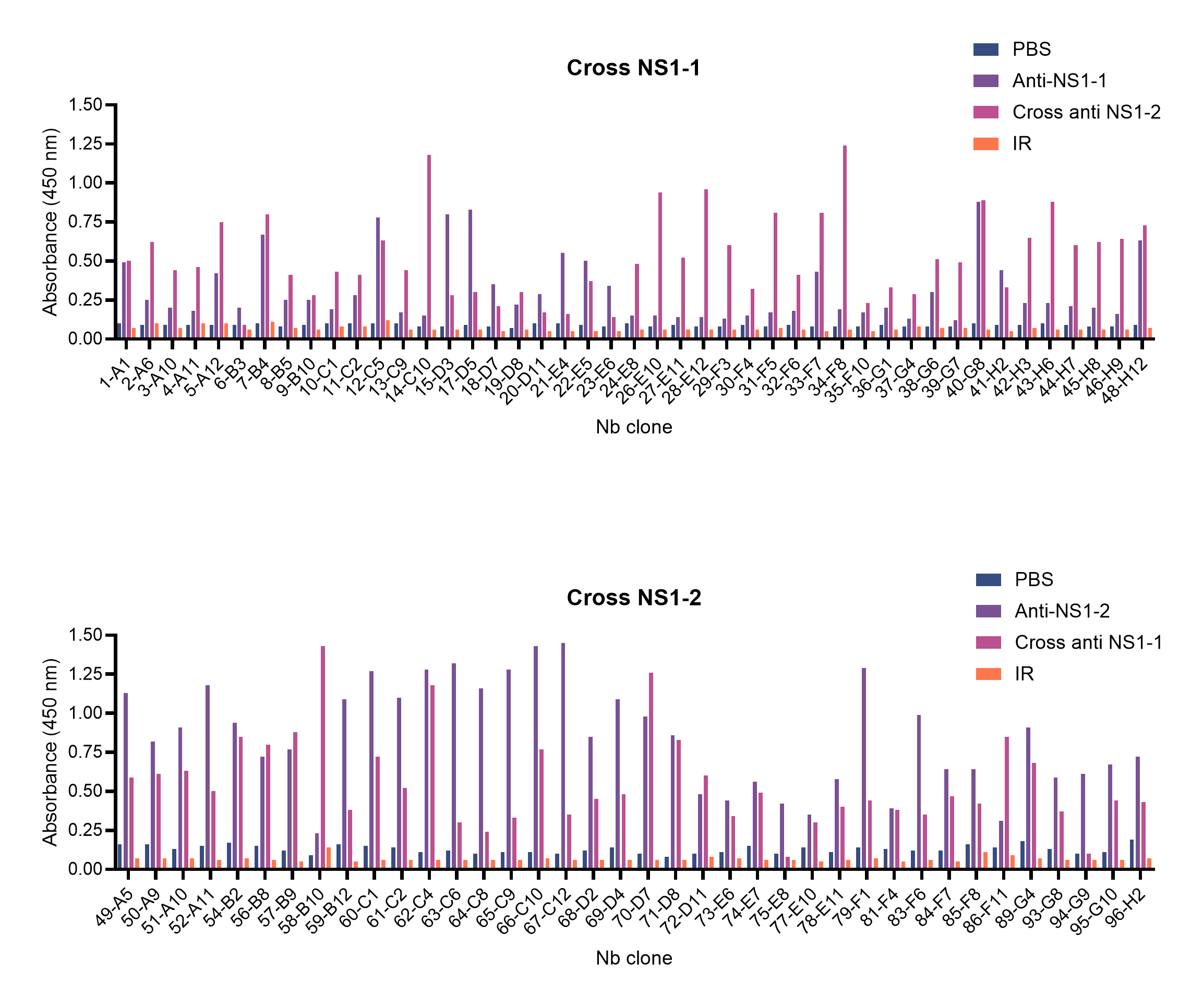

Supplement: S2 Fig — In the upper panel, clones selected using NS1-1 were tested against NS1-1, NS1-2, non-coated wells (PBS or supernatant of non-transfected cells), and an irrelevant His-tagged protein. In the lower panel, clones selected with NS1-2 were tested against NS1-2, NS1-1, non-coated wells (PBS), and an irrelevant His-tagged protein. Several cross-reactive clones were identified. (TIF) [file pntd.0013168.s005.tif]

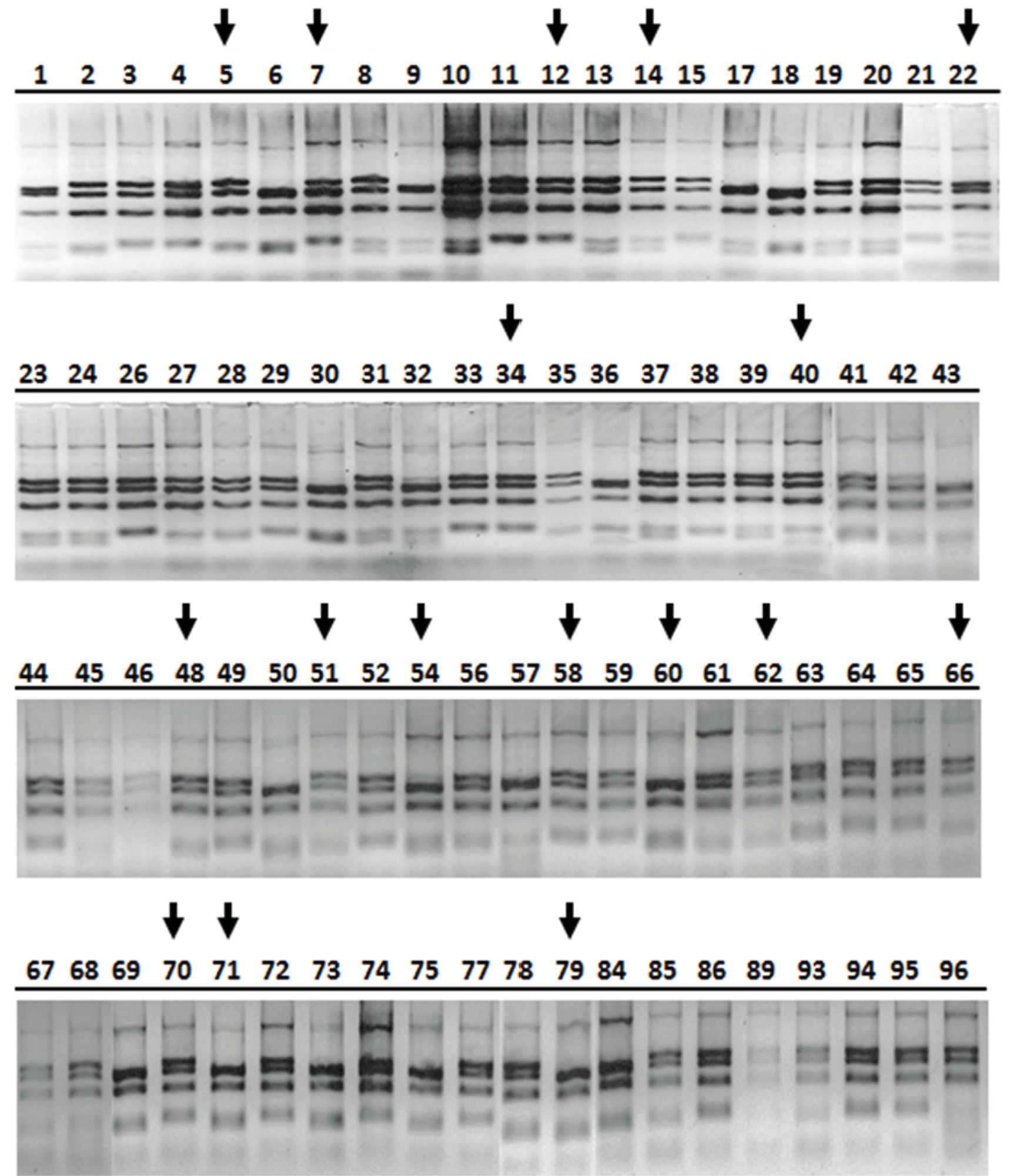

Supplement: S3 Fig — RFLP analysis showing patterns obtained after restriction digestion of PCR fragments with HinfI, a high frequency cutting restriction enzyme. Samples were analyzed on a 2.5% agarose gel and stained with ethidium bromide. At least 18 distinct patterns were identified (arrows). (JPG) [file pntd.0013168.s006.jpg]

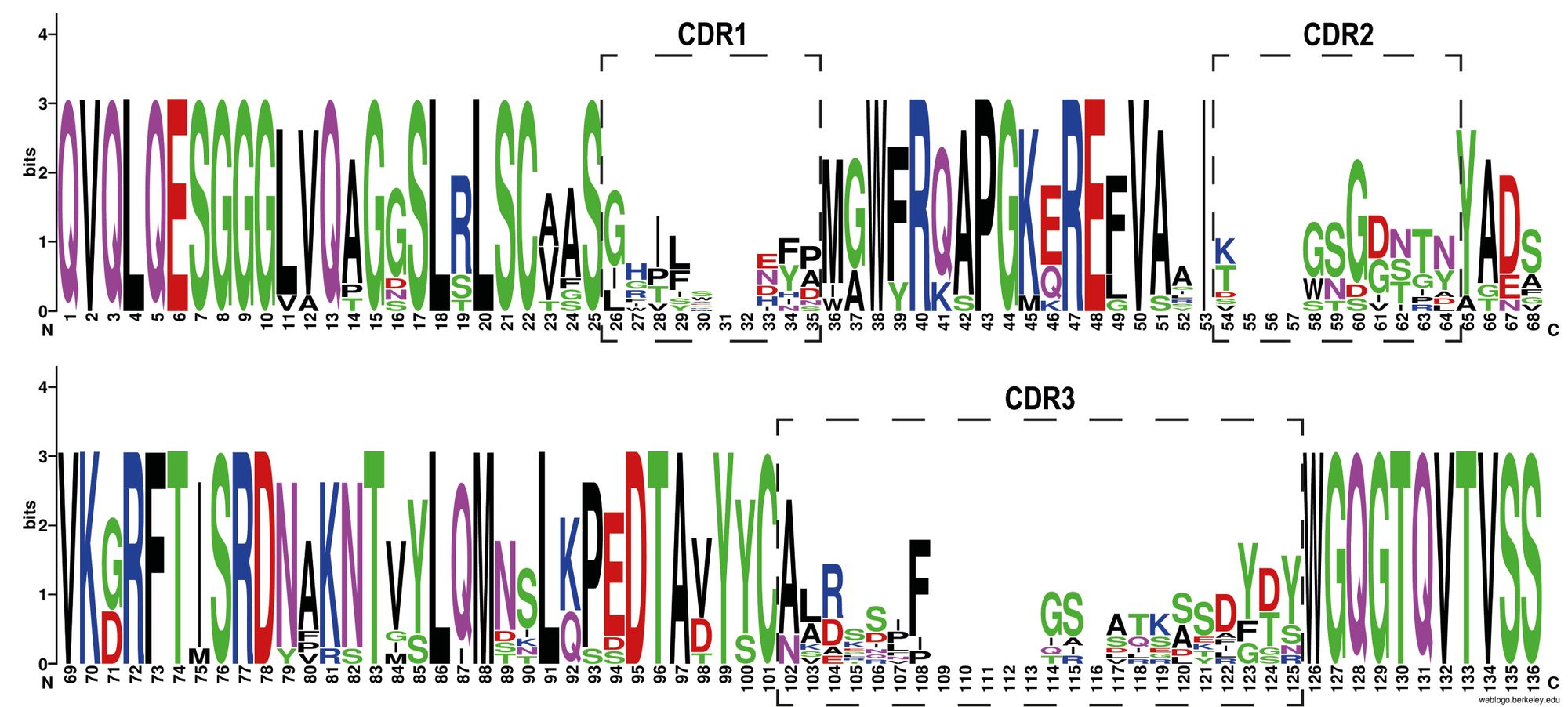

Supplement: S4 Fig — A sequence logo plot was generated using 11 unique Nb sequences with WebLog3, highlighting high variability in the CDR3 domains. (JPG) [file pntd.0013168.s007.jpg]

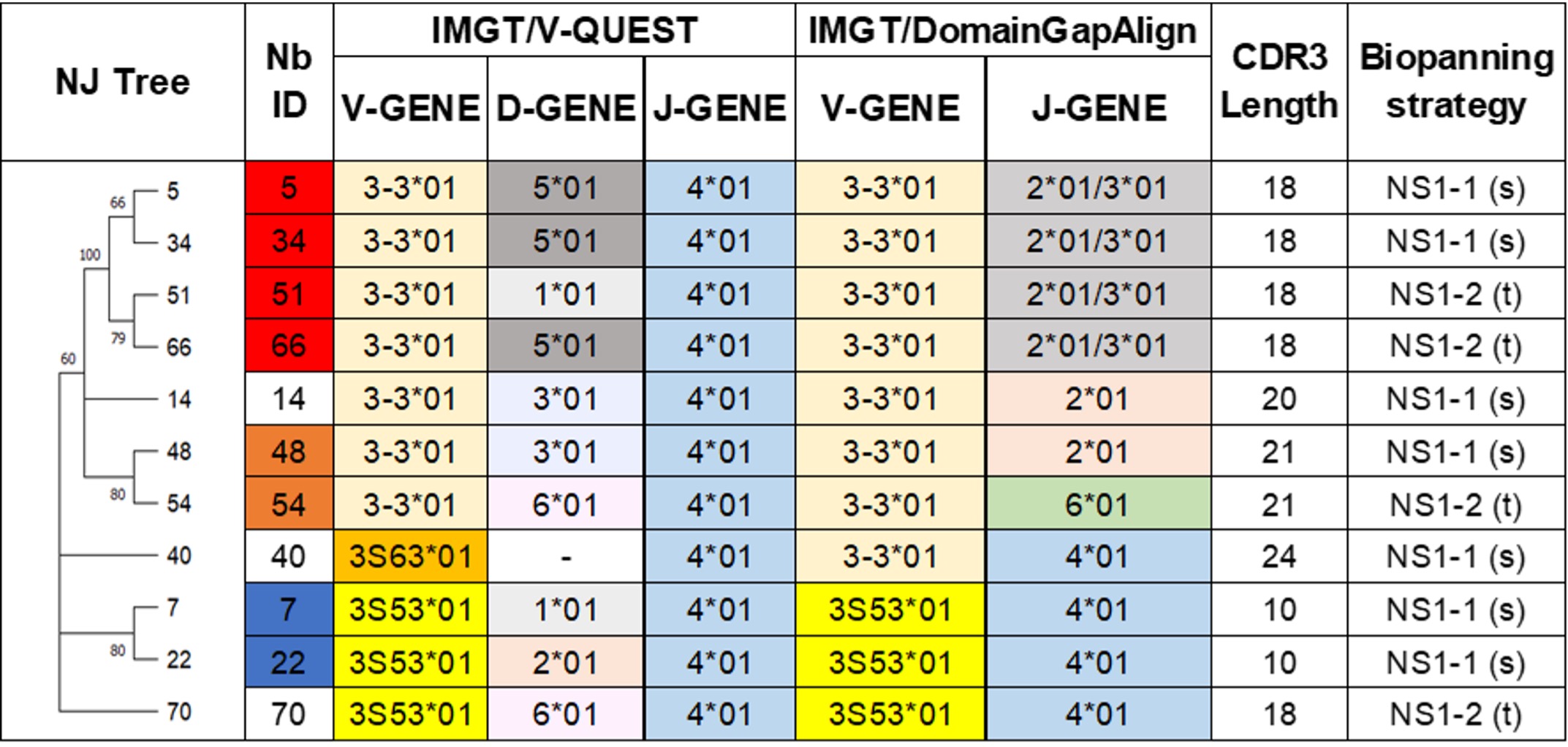

Supplement: S5 Fig — Phylogenetic analysis of Nbs selected against NS1-1 and NS1-2 identified three main clusters (red, orange, and blue). Group 1 (Nb5, Nb34, Nb51, and Nb66) exhibited an 18-amino-acid CDR3 and consistent V gene 3-301 usage, along with J genes 401 or 201/301. Group 2 (Nb48 and Nb54) featured a longer CDR3 (21 amino acids) and displayed variable J gene usage, as determined by DomainGapAlign. Group 3 (Nb7 and Nb22) had a compact 10-amino-acid CDR3 with consistent V gene 3S5301 and J gene 401 usage. Nanobodies Nb14, Nb40, and Nb70 did not cluster within these groups and instead displayed unique genetic and structural characteristics, with Nb40 having the longest CDR3 sequence among all analyzed variants. Germline origins were determined using IMGT/V-QUEST (columns 3–5) and IMGT/DomainGapAlign (columns 6–7), while CDR3 length and biopanning strategies are detailed in columns 8 and 9. (JPG) [file pntd.0013168.s008.jpg]

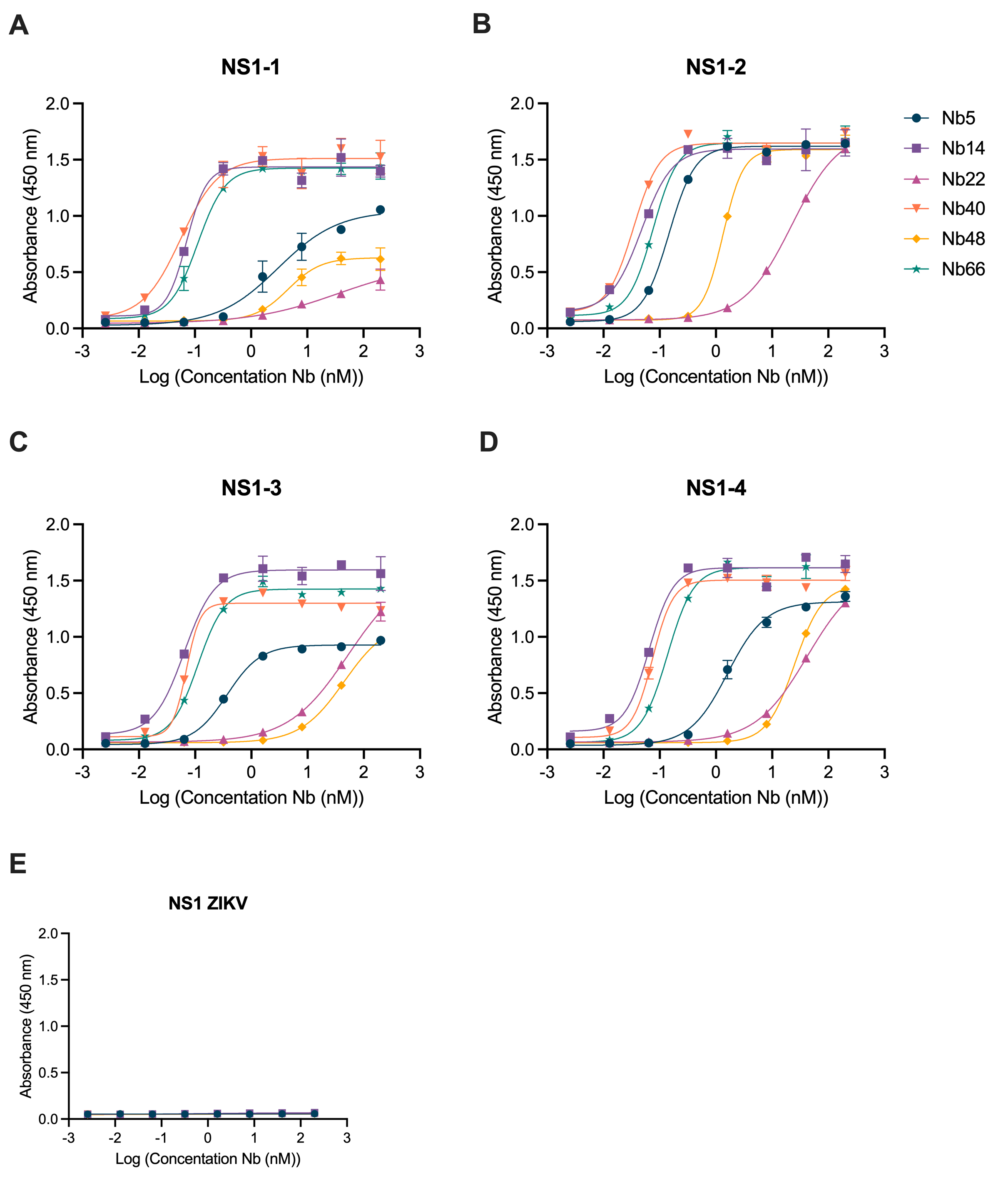

Supplement: S6 Fig — ELISA plates were coated with recombinant NS1 proteins and purified HRP-coupled Nbs serially diluted were added. The absorbance curves at 450 nm show the specific binding profile of each Nb to the different DENV NS1. None of the Nbs showed reactivity toward ZIKV NS1. (TIFF) [file pntd.0013168.s009.tiff]

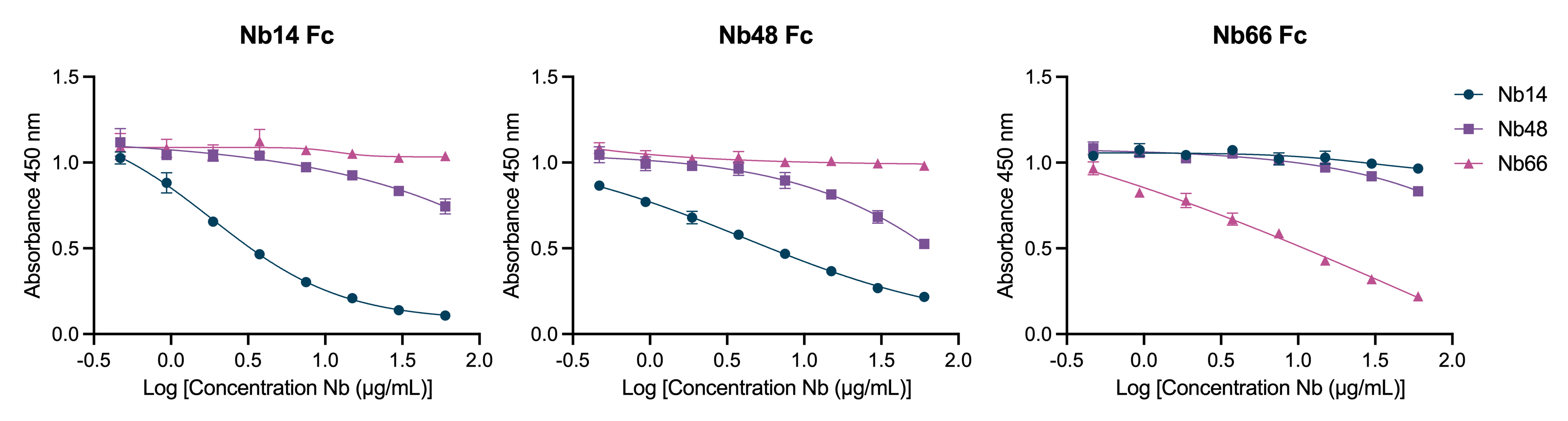

Supplement: S7 Fig — A competitive binding assay was conducted using Fc-fused Nbs and their monovalent counterparts to assess epitope overlap. The results indicate that Nb48 and Nb14 compete for the same binding site on the NS1 protein, suggesting recognition of a shared epitope. In contrast, Nb66 does not compete with either Nb48 or Nb14, indicating that it targets a distinct epitope. (TIFF) [file pntd.0013168.s010.tiff]

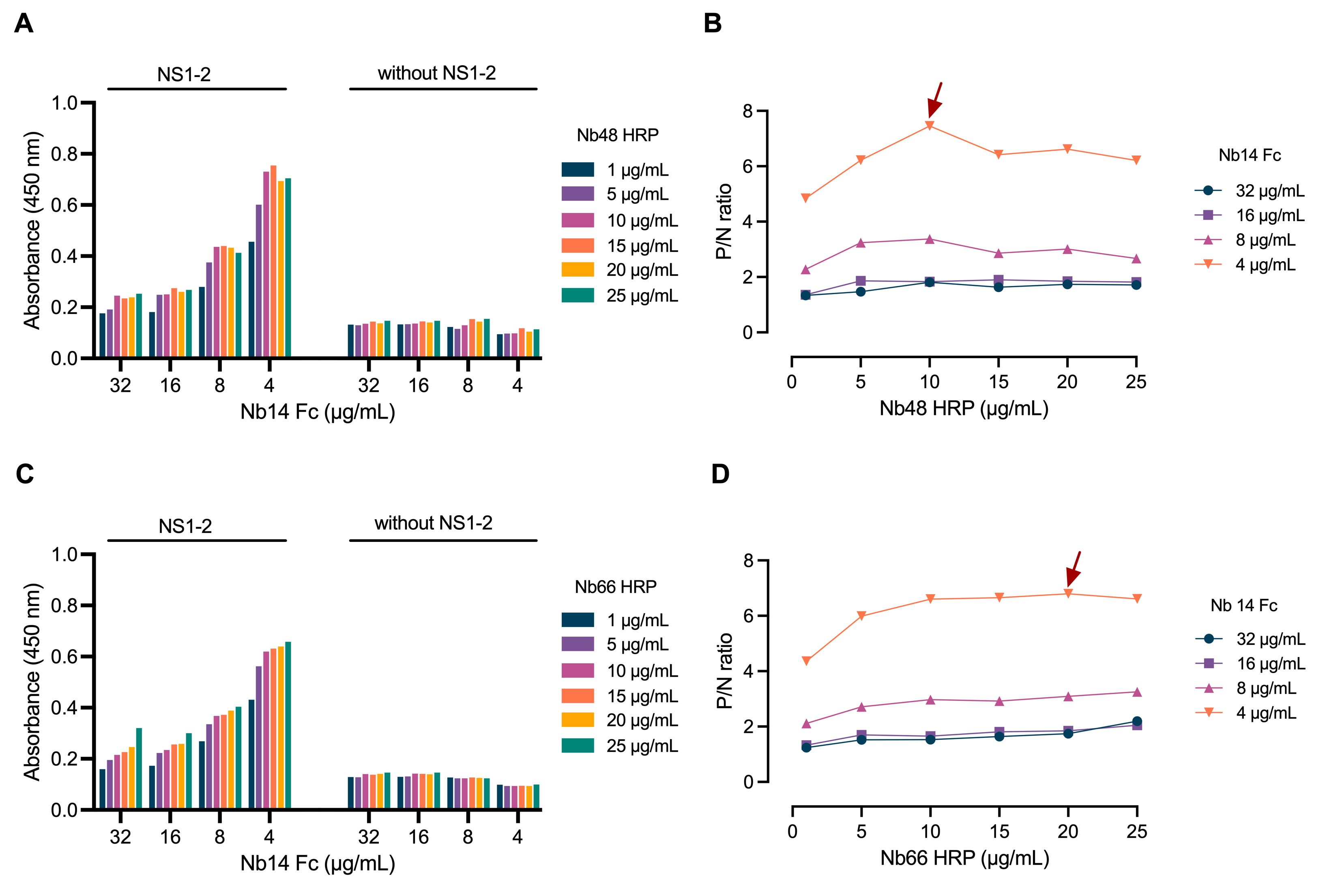

Supplement: S8 Fig — Absorbance values at 450 nm obtained from two-dimensional titration using different concentrations of Nb14 Fc as the capture antibody and Nb48 HRP (A) or Nb66 HRP (C) as the detection antibody. The assay was performed with and without 0.5 μg/mL of NS1-2. P/N ratio for combinations of Nb14 Fc and Nb48 HRP (B) or Nb66 HRP (D), indicating the best combination for the highest P/N ratio. The P/N ratio is calculated by dividing the absorbance of positive samples (NS1-2) by the absorbance of negative samples (without NS1-2). (TIFF) [file pntd.0013168.s011.tiff]

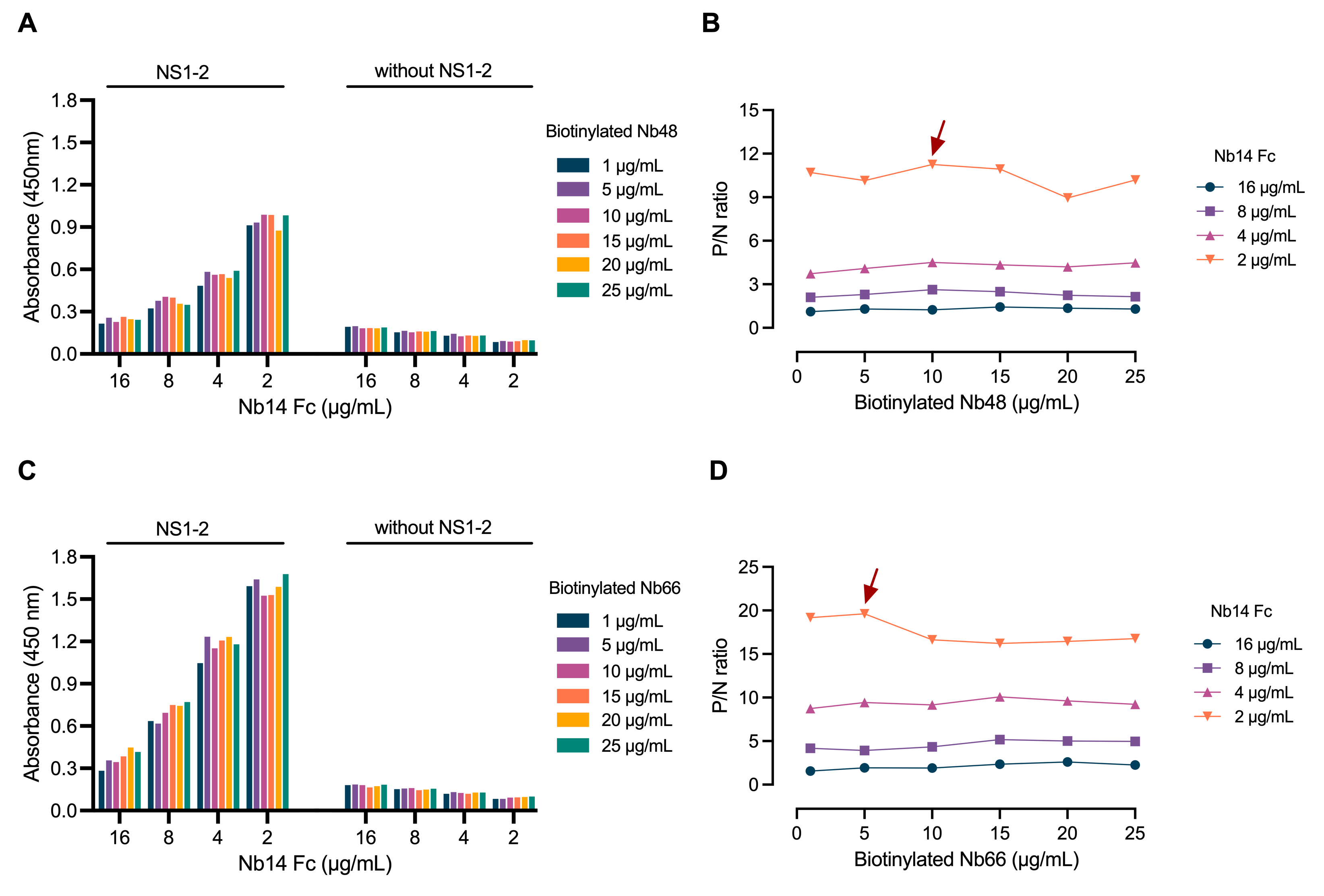

Supplement: S9 Fig — Absorbance values at 450 nm obtained from two-dimensional titration using different concentrations of Nb14 Fc as capture antibody and biotinylated Nb48 (A) or biotinylated Nb66 (C) as the detection antibody. The assay was performed with and without 0.5 μg/mL of NS1-2. P/N ratio for combinations of Nb14 Fc and biotinylated Nb48 (B) or biotinylated Nb66 (D), indicating the best combination for the highest P/N ratio. The P/N ratio is calculated by dividing the absorbance of positive samples (NS1-2) by the absorbance of negative samples (without NS1-2). (TIFF) [file pntd.0013168.s012.tiff]

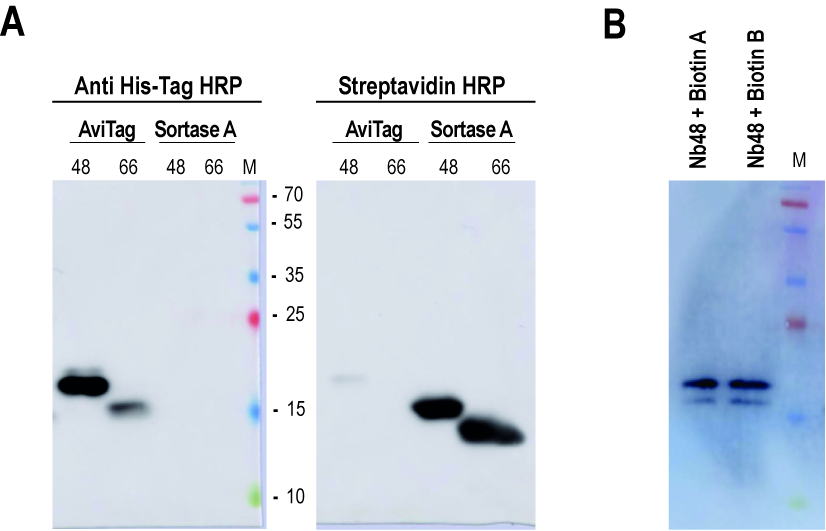

Supplement: S10 Fig — Nanobodies were biotinylated using either the AviTag system or the sortase A system. A-The left panel shows a western blot probed with an anti-His-HRP antibody, detecting His-tagged Nbs biotinylated via the AviTag system, which retains the His tag during biotinylation. No signal was observed for Nbs biotinylated with the sortase A system, as the His tag is removed during the reaction. The right panel presents a western blot of the same samples detected with Streptavidin-HRP, showing a weak signal for Nbs biotinylated via the AviTag system and a stronger signal for those biotinylated using the sortase A system. B-Western blot using Streptavidin-HRP to detect Nbs biotinylated via the AviTag system using A: commercial D-(+)-Biotin (catalogue 2031, Sigma-Aldrich) or B: Fast Dissolve Biotin 10.000 mcg, vitamin supplement (Carlyle). (TIF) [file pntd.0013168.s013.tif]

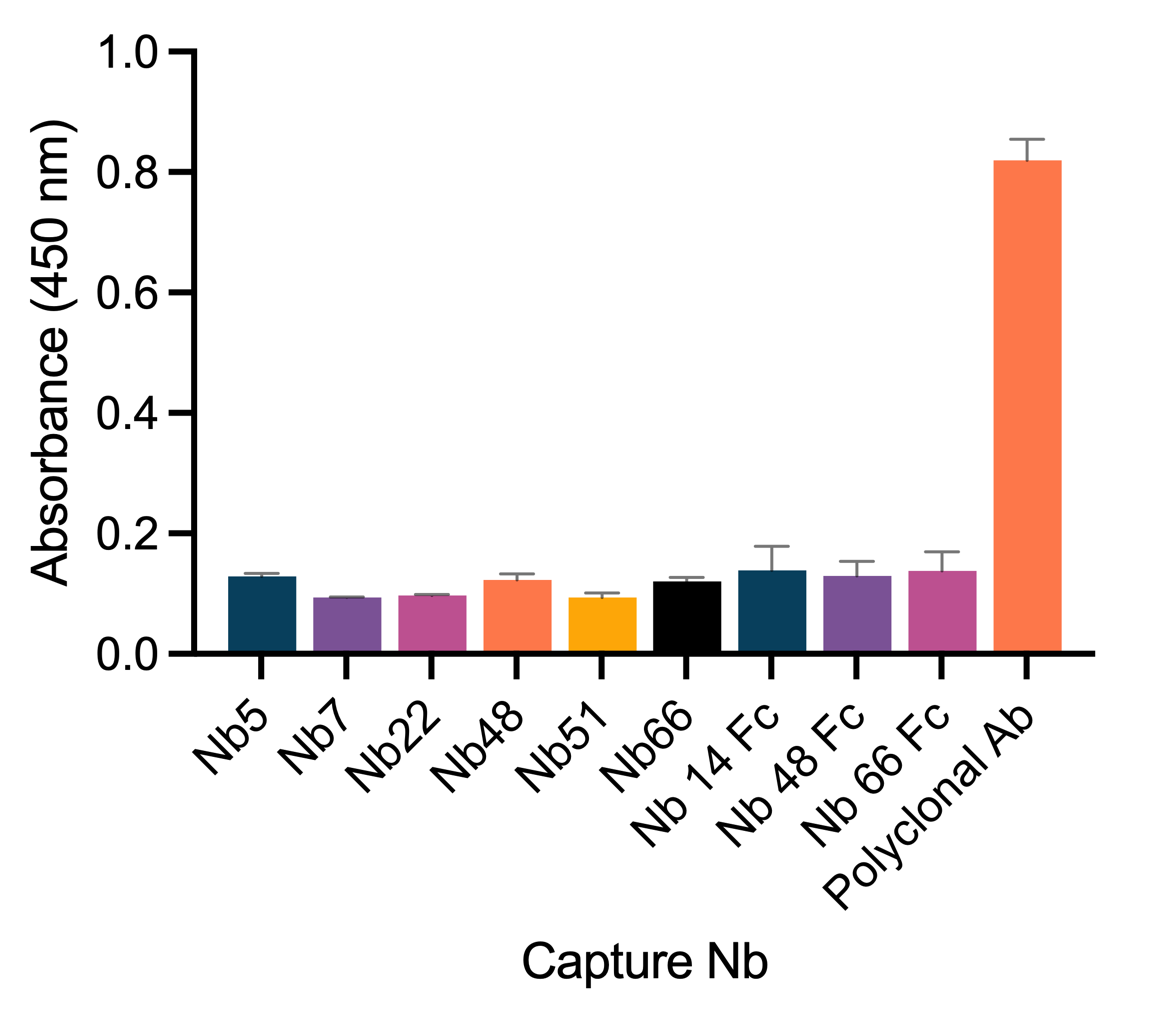

Supplement: S11 Fig — The ability of Nbs, in both monovalent and Fc-fused formats, to capture NS1 from clinical serum samples was assessed using the established cNb-ELISA protocol. None of the tested Nbs demonstrated detectable NS1 capture under the conditions employed. (TIFF) [file pntd.0013168.s014.tiff]

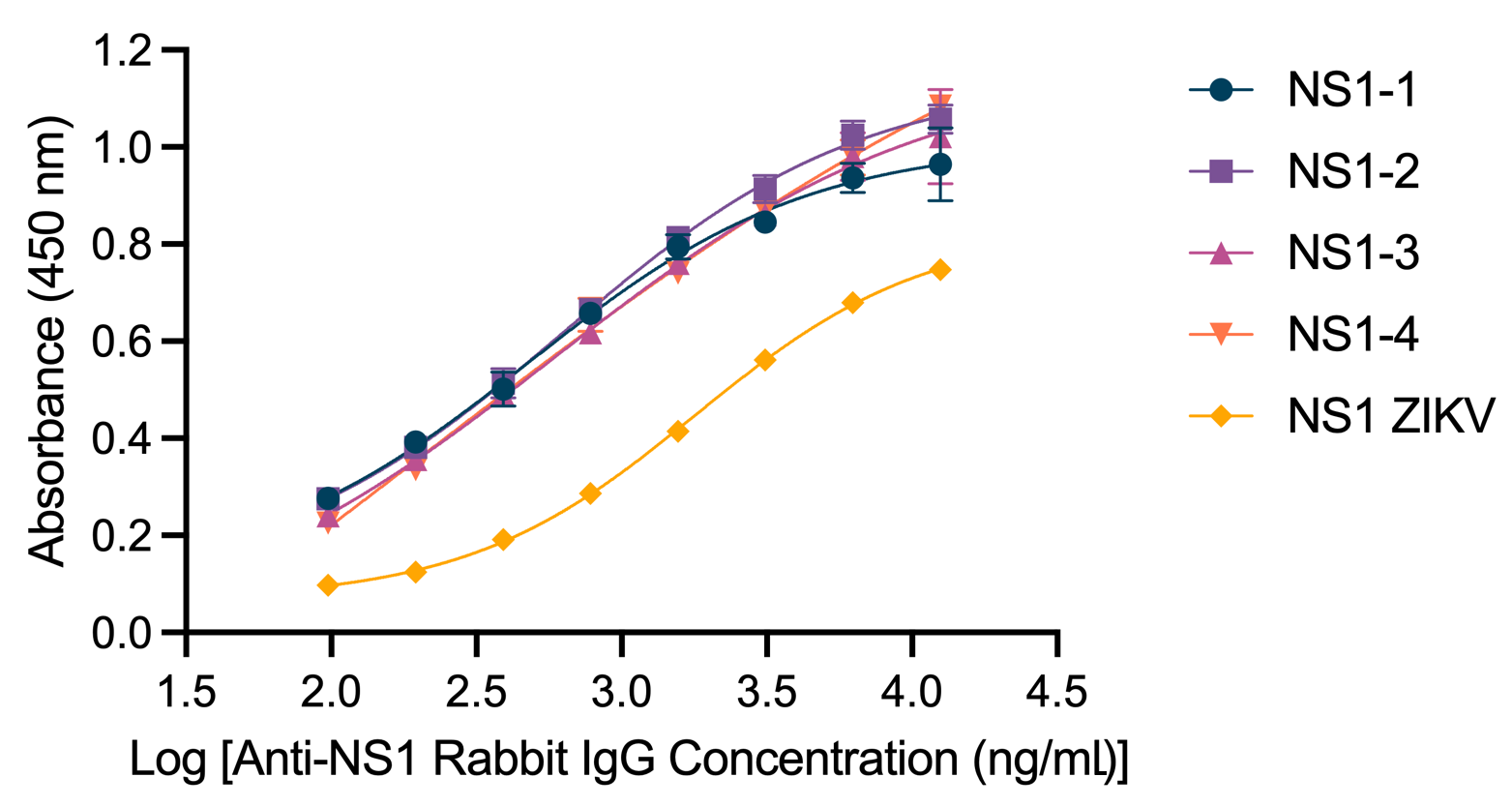

Supplement: S12 Fig — Purified rabbit IgG obtained after a five-dose immunization protocol was serially diluted to detect NS1 proteins (2 μg/mL). Specific recognition was detected using an anti-rabbit HRP-coupled antibody. Each experiment was conducted in duplicate. (TIF) [file pntd.0013168.s015.tif]

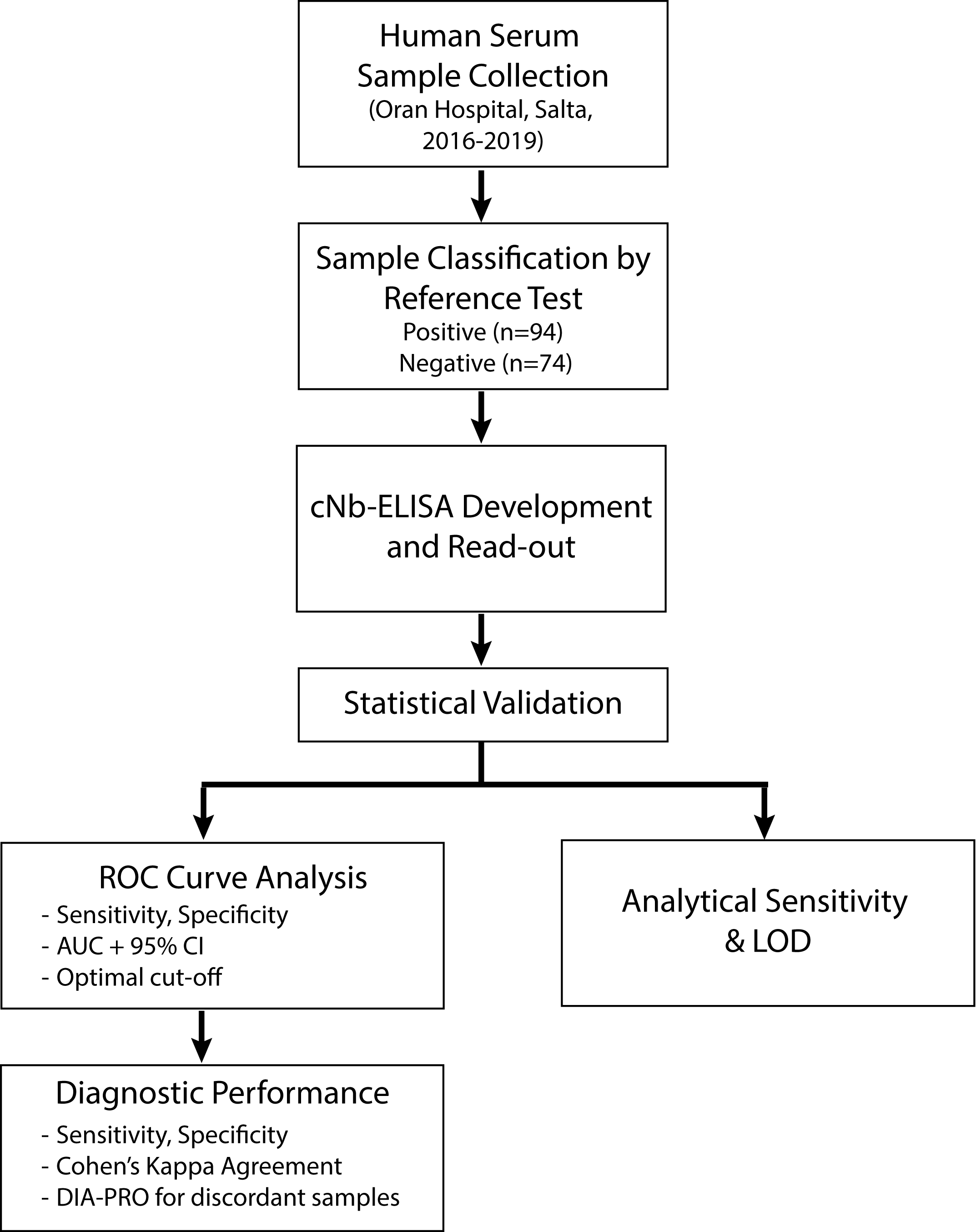

Supplement: S13 Fig — Human serum samples were collected at Orán Hospital, Salta (2016–2019) and classified by a reference diagnostic test (positive, n=94; negative, n=74). Samples were analyzed using the cNb-ELISA, and results were subjected to statistical validation. Diagnostic validation included ROC curve analysis (sensitivity, specificity, AUC, and cut-off determination), followed by assessment of diagnostic performance (sensitivity, specificity, Cohen’s kappa agreement, and DIA-PRO analysis for discordant samples). Analytical validation included determination of sensitivity and limit of detection (LOD). (TIF) [file pntd.0013168.s016.tif]
