# Supplementary material for: Engineered Nanobodies for early and accurate diagnosis of dengue virus infection
Source: PLoS Negl Trop Dis. 2025 Oct 31;19(10):e0013168. doi: 10.1371/journal.pntd.0013168 (PMC12591456; doi:10.1371/journal.pntd.0013168)
Supplement: Attachment — Submitted filename: Response to reviewers.docx [file pntd.0013168.s018.docx]

**RESPONSES TO EDITOR AND REVIEWERS**

**Additional Editor Comments:**

Your manuscript was reviewed by 3 independent reviewers and an editorial board member. While the reviewers appreciate the presented work, major revision will be needed for the manuscript to be considered for publication in PLoS NTDs. Several issues were raised about the use of a polyclonal serum to generate a capture reagent, epitope specificity of the Nbs, the analysis of diagnostic accuracy, and cross-reactivity with other flaviviruses. In addition, the reviewers provided comments aimed at improving the manuscript’s background on NS1-based diagnostics, enhancing methodological detail for reproducibility, and reducing redundancy in the discussion section.

**Response:**

We are sincerely grateful for the reviewers’ thoughtful corrections and suggestions, which have significantly contributed to improving the quality of our manuscript. All comments have been carefully considered, additional experiments have been conducted, and the text and figures have been revised accordingly

**Journal Requirements:**

1) We ask that a manuscript source file is provided at Revision. Please upload your manuscript file as a .doc, .docx, .rtf or .tex. If you are providing a .tex file, please upload it under the item type u2018LaTeX Source Fileu2019 and leave your .pdf version as the item type u2018Manuscriptu2019.

2) We noticed that you used the phrase 'data not shown' in the manuscript. We do not allow these references, as the PLOS data access policy requires that all data be either published with the manuscript or made available in a publicly accessible database. Please amend the supplementary material to include the referenced data or remove the references.

- Line 477 remove ‘data not shown’

- Line 652 remove ‘data not shown’

- Line 689 remove ‘data not shown’

**Response:**

- Line 477 remove ‘data not shown’: has been removed

- Line 652 remove ‘data not shown’: has been removed, new S10B Fig has been added (now **line 748**).

- Line 689 remove ‘data not shown’, has been removed.

3) We do not publish any copyright or trademark symbols that usually accompany proprietary names, eg ©, ®, or TM (e.g. next to drug or reagent names). Therefore, please remove all instances of trademark/copyright symbols throughout the text, including:

-TM on pages: 11, and 19.

**Response:**

Proprietary names were removed.

4) Thank you for including an Ethics Statement for your study. Please include:

i) A statement that formal consent was obtained (must state whether verbal/written) OR the reason consent was not obtained (e.g. anonymity). NOTE: If child participants, the statement must declare that formal consent was obtained from the parent/guardian.].

**Response:**

We requested this information from the Oran Hospital, which holds the sample bank. A note from the director of the Tropical Diseases Laboratory responding to the request is attached.

The following text has been added in Methods:

**Lines 349-350:** *Anonymized human serum samples (2016-2019) were taken at the Laboratory of Tropical Diseases, San Vicente de Paul Hospital, Orán, Salta, Argentina.*

The letter has been added as S13 Fig, as requested by the Editorial Manager.

**Line 1330:** Statement on Human Sample Anonymization.

5) We have noticed that you have uploaded Supporting Information files, but you have not included a complete list of legends. Please add a full list of legends for your Supporting Information files (Supplementary Tables) after the references list.

**Response:**

The requested information has been added:

**Line 1234:** Table S1. *List of primers used in this study*.

**Lines 1236-1237:** Table S2. *Discordant results between Platelia Dengue NS1 Ag kit (BioRad) from Oran Hospital, DIA.PRO kit and cNb-ELISA*.

**Lines 1239-1241:** Table S3. *Sample OD, cutoff and result interpretation for every sample used in this study according to the Platelia Dengue NS1 Ag kit (BioRad) from Oran Hospital, the developed cNb-ELISA or DIA.PRO kit.*

**Reviewers' Comments:**

Reviewer's Responses to Questions

Key Review Criteria Required for Acceptance?

As you describe the new analyses required for acceptance, please consider the following:

**METHODS**

-Are the objectives of the study clearly articulated with a clear testable hypothesis stated?

-Is the study design appropriate to address the stated objectives?

-Is the population clearly described and appropriate for the hypothesis being tested?

-Is the sample size sufficient to ensure adequate power to address the hypothesis being tested?

-Were correct statistical analysis used to support conclusions?

-Are there concerns about ethical or regulatory requirements being met?

**Reviewer #1:** 1. The methodology primarily focused on optimizing the ELISA conditions (e.g., antibody concentrations and formats) through chessboard titration experiments, rather than employing detailed epitope mapping. The study tested different combinations of nanobodies as capture and detection antibodies to achieve optimal assay performance but did not utilize advanced methods such as epitope binning or structural analysis to precisely identify the binding sites of each nanobody on the NS1 antigen. While the ELISA was optimized for sensitivity and specificity, the lack of detailed epitope identification is a methodological limitation that could be addressed in future work to further enhance assay design (Page 14).

**Response:**

Regretfully we did not have access to advanced methods such as structural analysis at the moment we performed the experiments and wrote the manuscript. However, we have recently established a collaboration with Dr. Luo Dahai (Nanyang Technological University, Singapore) who is interested in obtaining that information. We hope to include this information in a future work that we will do in collaboration.

To provide information on Nbs binding we performed a competition experiment, and we added the following information on the text:

In Methods:

**Lines 315-322:** *To evaluate potential epitope overlap among Nb14, Nb48, and Nb66, a competitive ELISA was conducted using unlabeled monovalent Nbs as competitors and their corresponding Fc-fusion formats (Nb-Fc) as detection probes. Ninety-six-well ELISA plates were coated overnight at 4 °C with NS1-2 protein (2 µg/mL), followed by blocking with 3% (w/v) skim milk in PBST for 1 hour at room temperature. Serial two-fold dilutions of the unlabeled Nbs (starting at 60 µg/mL) were incubated with a fixed concentration of Nb-Fc (0.4 µg/mL) and added to the wells for 1 hour. After washing, bound Nb-Fc was detected using an HRP-conjugated anti-mouse antibody (1:5,000 dilution). Signal development was carried out using TMB substrate as previously described.*

In Results:

**Lines 435-439:** *To identify shared epitopes among the different Nbs under investigation, we performed a competition assay. In each reaction, we combined Fc-tagged Nbs—whose binding was detected using an anti-mouse antibody—with monovalent Nbs. This approach allowed us to determine that Nb48 and Nb14 compete for the same binding site on the NS1 protein, whereas the epitope recognized by Nb66 is located elsewhere on the protein (S7 Fig).*

S7 Fig has been added. Figure legend:

**Lines 1285-1289:** *S7 Fig: Identification of shared epitopes recognized by DENV NS1-specific Nanobodies. A competitive binding assay was conducted using Fc-fused nanobodies and their monovalent counterparts to assess epitope overlap. The results indicate that Nb48 and Nb14 compete for the same binding site on the NS1 protein, suggesting recognition of a shared epitope. In contrast, Nb66 does not compete with either Nb48 or Nb14, indicating that it targets a distinct epitope.*

2. The Methods section does not provide extensive details on the use of controls or replicates in some assays, which may affect the reproducibility and robustness of the results (Page 2).

**Response:**

The following information was added regarding controls and replicates:

**Line 296-297:** *An irrelevant Nb (a Nb specific for the receptor binding domain of SARS-CoV-2) was used as negative control. Added in Fig 1B.*

**Lines 300-301:** ...*as antigen.* *Detection of NS1 was assessed as described in the former assay. These experiments were performed in triplicates.*

**Lines 309-31****1:** *A monoclonal Anti-Dengue virus NS1 protein antibody (BEI resources NR-10121) and an irrelevant Nb were also serially diluted in a 5-fold dilution series and used as positive and negative controls, respectively.*

Data was added on table 1.

**Lines 313:** *from triplicate measurements.*

**Line 333:** *The assay was run with duplicate wells for each sample.*

**Line 343:** *in triplicate.*

3. The introduction briefly mentions NS1 as a biomarker but does not elaborate on its structural forms, secretion dynamics, or variability among the four DENV serotypes, all of which are important for understanding the challenges in developing broadly reactive diagnostic tools (Page 6).

**Response:**

We appreciate these suggestions; the text has been modified to incorporate information on structural forms, secretion dynamics, and variability of NS1 proteins among the four DENV serotypes. The following text has been added:

**Lines 107-119**: *NS1 is a 46–50 kilodalton (kDa) glycoprotein that displays high amino acid and nucleotide homology across multiple flaviviruses [12,13]. Sequence similarity among NS1 proteins from the four dengue virus serotypes ranges between 68% and 80%. In comparison, NS1 shares approximately 54% identity with Zika virus (ZIKV), 51–53% with Japanese encephalitis virus (JEV), and 49–56% with West Nile virus (WNV) [13]. The NS1 protein is assembled into multiple oligomeric forms. The dimeric form, predominantly found within host cells, facilitates interactions with host proteins and plays a critical role in viral replication. According to several reports, secreted NS1 can also exist in both hexameric and tetrameric forms, which contribute significantly to viral replication, immune evasion, and pathogenesis [14,15]. This protein es considered a potent early biomarker for dengue virus detection, given its secretion into the bloodstream during the acute phase of infection. It is detectable from day 1 of fever onset and remains present for up to 7–9 days in most patients, although it has been detected as late as day 18 [16].*

4. The article does not provide detailed information regarding the use of transfected Vero cells for screening. Although the Methods mention the use of recombinant protein produced in transfected cells for nanobody selection, there is no thorough explanation of the screening process involving these cells. This lack of detail may affect reproducibility and understanding of how well the selected nanobodies recognize the native NS1 protein in a cellular context.

**Response:**

A detailed protocol on Vero cells transfection for biopanning has been added:

**Lines 181-187:** *DENV-2, cloned into the pCAGGS-ss vector, was expressed in Vero cells for biopanning experiments. Transfection was carried out on a 96-well plate using Lipofectamine 2000. Each well was seeded with 1.5 × 10⁴ Vero cells. After 24 hours, 60 ng of plasmid DNA, diluted in 50 µL of Opti-MEM, was mixed with 0.2 µL of Lipofectamine 2000 (also diluted in 50 µL of Opti-MEM). The mixture was incubated at room temperature for 25 minutes to allow complex formation. Transfection complexes were then added to the cells, which were incubated for 48 hours at 37°C in a 5% CO₂ incubator to enable protein expression.*

A thorough explanation of the screening process is also included:

**Lines 208-226:** *Welles prepared for both selection strategies were incubated at room temperature for 1 hour with 2% skimmed milk in PBST. Recombinant phages were then subjected to three successive rounds of counter-selection, each involving a 15-minute incubation at room temperature of 200 µL of phage suspension (at a concentration of 10¹² phages/mL) in wells containing protein extracts derived from non-transfected HEK293T or Vero cells, as appropriate. Counter selection steps were included to diminish non-specific phage binding, as the llama was immunized with a supernatant containing several irrelevant proteins. After the final counter-selection step, 100 µL of the collected phage suspension was transferred to the selection wells containing either the recombinant NS1-1 protein or transfected Vero cells. The remaining 100 µL was added to wells containing protein extracts from non-transfected HEK293T or Vero cells, serving as negative controls. Plates were incubated for 2 hours at room temperature. Non-specifically absorbed phages were removed by increasing the number of washes with PBST after each round of panning. Bound phages were eluted a two-step elution process, using trypsin solution (T-elution) and exponentially growing TG1 cells (C-elution) as previously published [47]. The first elution step involved the incubation for 30 minutes at room temperature with 100 µL per well of TPCK-treated trypsin (0.25 mg/mL in PBS). Enzymatic activity was quenched by adding 5 µL per well of AEBSF protease inhibitor (4 mg/mL). A second elution step consisted of the incubation at 37°C for 30 minutes with 200 µL of exponentially growing E. coli TG1 cells.*

**Reviewer #2**: Yes to all the questions.

**Reviewer #3**: - Overall, a logical flow was followed for generation and selection of Nb, and for the selection of best performing clones for capture and detection of NS-1

- The NS1 protein used to immunize the llama is produced in insect cells (C6/36), which can represent a limitation because there is no clear consensus in the literature whether the NS1 produced by insect cells has the same characteristics as the one produced by mammalian cells. This should at least be discussed by the authors.

**Response:**

We appreciate the reviewer’s insightful observation regarding the potential limitations of using NS1 protein produced in insect cells (C6/36) for immunization. While it is true that glycosylation patterns differ between mammalian and insect cells, multiple studies have demonstrated that NS1 glycoproteins from both hosts are secreted and exist in various oligomeric forms (10.1038/s41467-022-34415-1; 10.3389/fimmu.2023.1205002; 10.1073/pnas.1017338108). Furthermore, NS1 has been successfully detected in the supernatant of C6/36 cells infected with various DENV serotypes using commercial ELISA kits (doi:10.1186/1743-422X-5-51; doi:10.1038/s41598-017-16231-6; doi:10.1007/s00705-012-1525-2), suggesting that NS1 produced in insect cells shares relevant epitopes with its mammalian counterpart.

It is also important to note that NS1 secretion kinetics vary across cell types and flaviviruses. For example, Alcalá et al. (10.1016/j.virol.2015.11.020) reported early NS1 secretion in C6/36HT cells infected with DENV-2 and DENV-4, while Ludert et al. (10.1186/1743-422X-5-51) observed a 12-hour delay in secretion compared to Vero cells. Ricciardi-Jorge et al. (10.1038/s41598-017-16231-6) found slower NS1 secretion in C6/36 cells infected with YFV, and Viloche Morales et al. (10.1038/s41598-024-52123-2) reported low levels of secreted NS1 despite high intracellular production in ZIKV-infected C6/36 cells.

In our study, NS1 was clearly present in the supernatant of C6/36 cells infected with all four DENV serotypes as immunization with this purified supernatant successfully elicited a specific immune response against NS1, enabling the selection of targeted antibodies.

We have added a discussion of this point to the revised manuscript to address the reviewer’s concern:

**Lines 717-726:** *In this study a llama was immunized with NS1 protein derived from insect cells (C6/36). Multiple studies have demonstrated that NS1 glycoproteins from insect and mammalian hosts are secreted and exist in various oligomeric forms [12,14,73]. Furthermore, NS1 has been reliably detected in the supernatant of C6/36 cells infected with different DENV serotypes using commercial ELISA kits [74–76], suggesting the presence of conserved epitopes. Secretion kinetics vary across cell types and flaviviruses, in the case of DENV, Alcalá et al. demonstrated that C6/36HT cells begin secreting soluble NS1 from DENV-2 and DENV-4 as early as 6 hours post-infection [77]. Ludert et al. observed similar secretion kinetics in C6/36HT cells, albeit with a 12-hour delay compared to Vero cells [74]. Or results confirm that NS1 was secreted into the supernatant following infection of C6/36 cells with DENV as immunization with this material successfully elicited a specific immune response, supporting its suitability for antibody generation despite the host origin.*

- Why was panning done only against NS1 from Dengue-1 and Dengue-2, if the aim was to generate cross-reactive Nb?

**Response:**

Although all four dengue virus serotypes have circulated in Argentina in recent years, DEN-1 and DEN-2 were the predominant strains. Therefore, we chose to begin Nbs selection using the NS1 protein from these two serotypes, a phrase added:

**Lines 407-410:** *Although all dengue serotypes have been reported to circulate in Argentina, we selected serotypes 1 and 2 for panning, as they have shown the highest frequency of circulation in the country in recent years [58–61].*

- Line 205: why was RFLP done, what is the benefit compared to the sequencing that was done?

**Response:**

We understand that sequencing is a far more powerful tool for determining which Nbs to use in subsequent studies. However, during the years these experiments were conducted, there were significant complications in transferring U.S. dollars out of the country. This made it very difficult to contract reliable sequencing services due to payment issues.

To address this, and to reduce the number of samples we needed to send for sequencing, we turned to RFLP analysis. This allowed us to examine a large number of clones using the tools available at the time. By doing so, we increased our chances of selecting clones with distinct patterns—likely corresponding to different sequences—for sequencing.

Our strategy proved effective: out of 96 clones analyzed by RFLP, only 15 were sent for sequencing, and we obtained 11 unique sequences, which became the basis for our work.

- Why do PSR sequence modified Nb have expression and purification issues? Can this be explained?

**Response:**

In our experience, it is common to encounter Nbs that express at adequate concentrations during small-scale production or screening steps, but whose yield drops significantly when scaled up. For this reason, we prefer to work with multiple clones initially and continue only with those that do not show expression issues during scale-up. In the specific case of monovalent PSR-Nbs constructs, as the monovalent PSW-Nbs did not show significant improvement as capture reagents compared with multivalent constructs, we decided not to spend time improving their yield and decided not to continue working with them.

There are several reports highlighting the difficulty of expressing Nbs (e.g., DOI: 10.1016/j.pep.2020.105645), and strategies have been proposed to improve expression, such as fusing them to maltose-binding protein (MBP) (e.g., DOI: 10.1016/j.pep.2013.07.001). Protocols have also been optimized for expressing low-yield Nbs when necessary (e.g., DOI [10.1016/j.pep.2021.105906](https://doi.org/10.1016/j.pep.2021.105906)).

Although we have not been able to find publications that analyze these issues in detail, some papers have suggested that poor expression may be due to failure in translocation from the cytoplasm, resulting in inefficient periplasmic extraction and purification. In our case, it is possible that the addition of the PSR sequence, which is rich in arginine, may be contributing to secretion problems.

The following has been added for clarification:

**Lines 478-482 :** *Additionally, due to scale-up challenges, the PSR-modified Nbs were not tested in our assay. While several strategies exist to address low expression yields—such as fusion to Maltose Binding Protein or protocols tailored for recalcitrant Nbs—we opted not to pursue yield optimization [62–64]. This decision was based on the lack of significant improvement in capture performance following the addition of the PSW polystyrene-binding peptide.*

**RESULTS**

-Does the analysis presented match the analysis plan?

-Are the results clearly and completely presented?

-Are the figures (Tables, Images) of sufficient quality for clarity?

**Reviewer #1**: 1. Inconsistent Notation: The difference in notation ("Nanobody 48" vs. "Nb66") likely reflects slight inconsistencies in writing, but both refer to specific nanobodies characterized and used in the assays (Line 428).

**Response:**

We only use the full term *Nanobody + number* at the beginning of a sentence. This was recommended by the person who reviewed the English in the manuscript. In all other cases, when *Nanobody + number* appears elsewhere in the sentence, we use the abbreviation *Nbnumber*.

2. The article lacks detailed schematic diagrams of the ELISA procedures, which could aid in understanding and reproducing the assay, particularly for the complex combined nanobody formats. Including schematic diagrams for each key step throughout the article is highly recommended. Visual aids such as diagrams of the ELISA procedure and the combined Nanobody ELISA (cNb-ELISA) would greatly enhance readers’ understanding by clearly illustrating the workflow, antibody interactions, and assay design. For example:

• A schematic of the standard Nanobody-based ELISA showing capture antibody coating, antigen binding, detection antibody incubation, and signal development would clarify the optimization steps described (Page 14).

• A detailed diagram of the combined Nanobody ELISA, highlighting the use of specific nanobodies as capture and detection antibodies, sample addition, and detection readout, would help explain the assay’s development and validation process ([14,118,654]; [15,36,432]).

• Including flowcharts or stepwise illustrations of the statistical validation methods (ROC curve analysis, cut-off determination) could also aid comprehension of the assay’s diagnostic performance evaluation (Page 15).

**Response:**

We appreciate this suggestion for clarification.

Figure 3 has been modified to add this information. A scheme of the standard Nanobody-based ELISA has been added as figure 3A. The following text was added:

**Lines 546-552*:*** *A-Schematic representation of the two proposed Nanobody-based ELISA systems. System 1: 4 µg/mL of Nb14 Fc as the capture antibody and 10 μg/mL Nb48 HRP as the detection antibody. System 2: 2 µg/mL of Nb14 Fc as the capture antibody and 5 μg/mL biotinylated Nb66 as the detection antibody. B-Reactivity of System 1 with NS1 variants from DENV serotypes 1–4, ZIKV NS1, and human sera. C-Reactivity of System 2 with the same antigen panel. Data is shown as mean absorbance at 450 nm ± SD (n=3). Black dashed line represents the mean OD of negative control samples plus 3 standard deviations.*

Figure 4 was modified to add this information. A detailed diagram of the combined Nanobody ELISA ass been added as figure 4A. The following text was added:

**Lines 596-603**: *A-Schematic representation of the combined Nanobody ELISA workflow. B-Detection of recombinant NS1 proteins from DENV and ZIKV in a negative serum matrix. C-Detection of NS1 in pooled human serum samples from DENV-positive and negative patients. Serum dilutions (1:5 and 1:10) were analyzed using biotinylated Nb66 as detector. D-Specificity assessment of the combined Nb ELISA using supernatants from Vero cells infected with different flaviviruses (DENV-1 to DENV-4, ZIKV, YFV) and the unrelated arbovirus CHIKV, which co-circulate in endemic regions and cause overlapping clinical symptoms. Mock-infected supernatant was used as a negative control.*

A flowcharts about statistic validation has been added as S14 Fig. The following text was added:

In Results:

**Lines 607-608:** *A flow diagram of the validation process for the cNb-ELISA is shown as supplementary information (S13 Fig).*

Figure legend:

**Lines 1332-1339:** *S13 Fig: Flow diagram of the validation process for the combined Nanobody ELISA.*

*Human serum samples were collected at Orán Hospital, Salta (2016–2019) and classified by a reference diagnostic test (positive, n=94; negative, n=74). Samples were analyzed using the cNb-ELISA, and results were subjected to statistical validation. Diagnostic validation included ROC curve analysis (sensitivity, specificity, AUC, and cut-off determination), followed by assessment of diagnostic performance (sensitivity, specificity, Cohen’s kappa agreement, and DIA-PRO analysis for discordant samples). Analytical validation included determination of sensitivity and limit of detection (LOD).*

3. The hospital’s algorithm used unusually low cutoff values in some tests, which led to false positives. This underscores the importance of carefully defining cutoff thresholds for the cNb-ELISA to strike an appropriate balance between sensitivity and specificity ([6,50-56]).

**Response:**

We agree on the importance of accurately defining cutoff values, which is why we have determined ours as precisely as possible. Ideally, a different set of samples using an alternative algorithm would be used, but obtaining enough samples for meaningful statistical analysis is challenging. Unfortunately, we only have access to the data provided by hospital technicians, who apply pre-established criteria to determine the positivity or negativity of the samples.

**Reviewer #2:** yes to all the questions

**Reviewer #3**: - The results are mainly clearly presented and explained with appropriate figures

- In some cases, the concentrations of the mAbs are expressed in nanomolar (nM) units, while in others, they are expressed in nanograms per milliliter (ng/mL). This may not be very clear for the reader.

**Response:**

We appreciate that the reviewer pointing out this inconsistency.

EC₅₀ values are commonly reported in molar units (nM), and we have therefore kept them in this format. To improve clarity and ensure consistency within the ELISA assays, we have now expressed all other antibody concentrations in µg/mL. The following changes have been introduced in the revised manuscript:

**Line 326-327:** *Microplates were coated overnight at 4°C with serial two-fold dilutions of Nb14 Fc, starting from either 4 or 2 µg/mL.*

**Lines 513-518:** *For Nb14 Fc/Nb48 HRP pair, the highest P/N ratio was achieved with 4 µg/mL capture antibody and 10 µg/mL detection antibody (S8B Fig). Similarly, for Nb14 Fc/Nb66 HRP, the optimal signal was observed with 4 µg/mL capture antibody and 20 µg/mL detection antibody, with a P/N ratio close to 6 (S8D Fig). Based on these results, the first system for NS1 detection, named system 1, was proposed: 4 µg/mL Nb14 Fc as capture antibody, 10 µg/mL Nb48 HRP as detection antibody, and 1% BSA as the blocking reagent.*

**Lines 522-530:** *When utilizing these modified Nbs, a trend observed with HRP-conjugated Nbs emerged, with higher absorbance values achieved at even lower Nb14 Fc concentrations (2 µg/mL; S9 Fig). The highest P/N ratio for the Nb14 Fc/Nb48 biotin pair was obtained using 2 µg/mL capture antibody and 10 µg/mL detection antibody (S9B Fig). For Nb14 Fc/Nb66 biotin pair, the optimal combination was 2 µg/mL capture antibody and 5 µg/mL detection antibody (S9D Fig). These results demonstrate that biotinylated Nbs enhance signal amplification and improve the sensitivity of the sandwich ELISA. Therefore, another system, named system 2, was proposed: 2 µg/mL Nb14 Fc as the capture antibody, 5 µg/mL Nb66 biotin as the detection antibody, and 1% BSA as the blocking reagent.* Supplementary figures 8 and 9 were modified.

- The authors determined the cross-reactivity of the nanobodies only against Zika virus. In case of commercialization, it will be helpful to know whether the assay can also be used in regions where other flaviviruses co-circulate

**Response:**

We conducted a new set of experiments using supernatants from Vero cells infected with the viral strains available to us during this period. The corresponding data are presented in Fig 4D. Details of these experiments have been added:

In Methods:

**Lines 165-170:** *For cross-reactivity assays, Vero CCL-81 cells were infected with DENV-1 to -4, Chikungunya virus (CHIKV), and Yellow Fever virus (YFV) at a MOI of 0.1. Cell culture supernatants were collected between days 3 and 7 p.i., clarified by centrifugation, filtered, and stored at −80 °C until use. Viral titers were quantified by plaque assay and ranged from 1 × 10⁶ to 1 × 10⁷ plaque-forming units (PFU) per milliliter. Viral strains were kindly provided by Dr. Alicia Mistchenko from the Laboratory of Virology, Hospital de Niños Dr. Ricardo Gutiérrez, Buenos Aires, Argentina.*

**Lines 365-368**: *The cNb-ELISA protocol was further employed to assess the specificity of the detection system against other circulating viruses. For this purpose, supernatants from Vero cells infected with DENV serotypes, ZIKV, CHIKV, and YFV were analyzed.*

In Results:

**Lines 579-583:** *To further evaluate the specificity of our detection system for dengue virus NS1 protein, we tested supernatants from Vero cells infected with DENV serotypes 1–4, ZIKV, CHIKV, and YFV. The selected Nbs showed no cross-reactivity with other flaviviruses, including YFV and Zika virus, nor with the alphavirus CHIKV, which presents clinical symptoms that closely resemble those of dengue (Fig 4D).*

Figure legend:

**Lines 599-603:** *D-Specificity assessment of the combined Nb ELISA using supernatants from Vero cells infected with different flaviviruses (DENV-1 to DENV-4, ZIKV, YFV) and the unrelated arbovirus CHIKV, which co-circulate in endemic regions and cause overlapping clinical symptoms. Mock-infected supernatant was used as a negative control.*

In Discussion:

**Lines 858-859:** *We successfully generated and characterized Nbs capable of detecting NS1 from all four DENV serotypes with minimal no cross-reactivity to ZIKV , CHKV or YFV (Fig. 4D).*

- It seems that no competitive clustering / epitope binning was done for the Nb selected, as this would allow to select pairs of Nb for capture/detection that do not target the same epitopes/regions. Is there a rationale for not including these type of experiments? Maybe this could be discussed in the discussion section.

**Response:**

We have added information in response to question 1 of Reviewer 1 (Methods). We repeat here the information added.

To provide information on Nbs binding we performed a competition experiment, and we added the following information on the text:

In Methods:

**Lines 315-322:** *To evaluate potential epitope overlap among Nb14, Nb48, and Nb66, a competitive ELISA was conducted using unlabeled monovalent Nbs as competitors and their corresponding Fc-fusion formats (Nb-Fc) as detection probes. Ninety-six-well ELISA plates were coated overnight at 4 °C with NS1-2 protein (2 µg/mL), followed by blocking with 3% (w/v) skim milk in PBST for 1 hour at room temperature. Serial two-fold dilutions of the unlabeled Nbs (starting at 60 µg/mL) were incubated with a fixed concentration of Nb-Fc (0.4 µg/mL) and added to the wells for 1 hour. After washing, bound Nb-Fc was detected using an HRP-conjugated anti-mouse antibody (1:5,000 dilution). Signal development was carried out using TMB substrate as previously described.*

In Results:

**Lines 435-439**: *To identify shared epitopes among the different Nbs under investigation, we performed a competition assay. In each reaction, we combined Fc-tagged Nbs—whose binding was detected using an anti-mouse antibody—with monovalent Nbs. This approach allowed us to determine that Nb48 and Nb14 compete for the same binding site on the NS1 protein, whereas the epitope recognized by Nb66 is located elsewhere on the protein (S7 Fig).*

S7 Fig has been added. Figure legend:

**Lines 1285-1289:** *S7 Fig: Identification of shared epitopes recognized by DENV NS1-specific Nanobodies. A competitive binding assay was conducted using Fc-fused nanobodies and their monovalent counterparts to assess epitope overlap. The results indicate that Nb48 and Nb14 compete for the same binding site on the NS1 protein, suggesting recognition of a shared epitope. In contrast, Nb66 does not compete with either Nb48 or Nb14, indicating that it targets a distinct epitope.*

- It is not clear what the benefit of this study / new Nb is compared to previous studies, like the following publication that seems to be more detailed, especially given that the analysis of cross-reactivity goes beyond ZIKA virus, which seems critical for the proposed diagnostic assay.

o Shriver-Lake, L.C., Liu, J.L., Zabetakis, D. et al. Selection and Characterization of Anti-Dengue NS1 Single Domain Antibodies. Sci Rep 8, 18086 (2018). <https://doi.org/10.1038/s41598-018-35923-1>

**Response:**

Our main goal in this study was to develop a low-cost detection system compatible with the equipment and technical expertise typically found in clinical laboratories. Given its widespread use, we selected ELISA as the core technique.

While previous studies have investigated NS1 detection using Nbs, a major limitation is the lack of validation with patient samples. For instance, Fatima et al. evaluated Nbs using recombinant NS1 from E. coli or inactivated viral supernatant from dengue serotype 2, but not clinical specimens. Similarly, Shriver-Lake et al. assessed cross-reactivity with other flaviviruses using serum spiked with recombinant protein and employed the Luminex MagPlex assay, which is not standard in our clinical settings. In contrast, our study shows that while our Nbs can detect recombinant NS1 spiked into serum, they do not capture native NS1 from infected patient samples. The NS1 proteins used—both dengue and other flaviviruses—were commercially produced in HEK cells by the Native Antigen Company, consistent with our expression system. Only Anderson et al. used human serum samples, but with just four per serotype—insufficient for meaningful statistical analysis. Moreover, their system failed to detect NS1 from DENV-2.

Thus, the key contribution of our work is demonstrating that Nbs can be applied to detect NS1 in actual patient samples, allowing the validation of the diagnostic system—a capability not previously reported.

To emphasize this, we added:

**Lines 714-715**: *A common limitation in prior studies is the use of recombinant proteins, spiked serum, or a limited number of infected human samples—insufficient for robust diagnostic validation.*

- Figure 1: overall, most Nb show very poor capture capacity in this assay. What was the background absorbance observed for capture using Nb that are not specific for NS1? Why was this control not added? Comparison with a non-specific Nb would allow to assess the Nb that show very low absorbance are actually specific in capturing the NS1.

**Response:**

We thank the reviewer for pointing out this oversight. The information regarding negative control has now been included in Figure 1. The signal observed for the irrelevant Nb is comparable to that of Nbs lacking capture capability.

The following clarification has also been added to the Methods section:

**Lines 296-297:** *An irrelevant Nb (specific for the receptor-binding domain of SARS-CoV-2) was used as a negative control.*

Line 402: “the fusion of the Fc domain to combine these benefits”. It is not clear why this Fc fusion is done. What do the authors mean with ‘these benefits’.

**Response:**

Polystyrene-binding peptides (Nb-PSW) enable surface orientation and bivalent Nbs improve binding through multimerization. The benefits we highlight for Fc-Nbs are: multimerization, which enhances binding avidity, and orientation, which improves antigen accessibility on plastic surfaces. Fc-Nbs incorporate two identical Nb units, and the Fc region facilitates favorable orientation during passive adsorption, improving detection efficiency.

Multimerization and proper orientation are well-documented strategies for enhancing diagnostic performance. For instance, Anderson et al. showed that oriented SpyTag-Nb dimers significantly outperformed monomers in detecting staphylococcal enterotoxin B and later applied a similar approach to improve NS1 detection. Additionally, several studies targeting the SARS-CoV-2 spike RBD protein have employed multimerized Nbs to boost binding and sensitivity (e.g., 10.1126/science.abe3255; 10.1038/s41594-020-0469-6; 10.7554/eLife.64815).

To clarify, the text has been modified:

**Lines 468-470:** *to combine these benefits of enhanced avidity and improved immobilization through Fc-mediated orientation on plastic surfaces.*

- The Fc fused Nb clearly is the most potent as capture antibody (figure 2). How can you explain this, as the bivalent Nb also has two binding sites.

**Response:**

Several studies have compared bivalent Nbs with Fc-fused formats, showing that Fc fusion generally enhances binding and functional activity, including neutralization (e.g., 10.1128/JVI.03178-13, 10.3389/fimmu.2022.893648). Anbuhl et al. (10.1016/j.bcp.2024.116457) reported superior performance of an Fc-coupled CXCR4-targeting Nb over its bivalent counterpart, not only in binding but also in receptor clustering.

They attributed this to the structural advantage of Fc fusion via the C-terminus, compared to the flexible GS-linker connecting two nanobody domains. Although the bivalent Nb can engage both paratopes simultaneously, its apparent affinity may be compromised by linker flexibility, increased molecular size, or altered kinetics.

Additional differences include the Fc domain’s rigidity and stability, which might improve avidity through better spatial orientation of binding sites. Fc fusion also supports proper folding and secretion in mammalian systems, whereas bivalent constructs with long linkers may misfold or aggregate, reducing functional performance.

The text has been rewritten for clarification as follows:

**Lines 732-743:** *Our results showed that multimerizing Nbs, either through Fc fusion or bivalent design, significantly enhanced their ability to capture recombinant NS1 protein. However, despite the increased affinity of bivalent constructs, their capture performance did not exceed that of Fc-fused Nbs (Fig. 2). Previous studies have consistently demonstrated that Fc fusion improves binding, neutralization, and overall functional activity [78,79]. Notably, Anbuhl et al. reported superior binding and receptor clustering with an Fc-coupled CXCR4-targeting Nb compared to its bivalent counterpart. This advantage was attributed to the structural rigidity of Fc fusion at the C-terminus, in contrast to the flexible GS-linker used in bivalent formats [80]. Although bivalent Nbs can engage both paratopes, their apparent affinity may be compromised by linker flexibility, increased molecular size, or altered kinetics. Importantly, bivalent constructs remain valuable for low-cost diagnostic platforms, as they can be efficiently produced in bacterial systems—offering a more economical alternative to Fc-fused Nbs, which require eukaryotic expression systems.*

- Figure 3 shows a dotted line, what does this represent, this should be explained in the figure legend

**Response:**

We thank the reviewer for identifying this mistake. The missing information has been added to the text as follows:

**Lines 551-552:** *Black dashed line represents the mean OD of negative control samples plus 3 standard deviations.*

- The authors use a polyclonal serum as a capture antibody for the final Elisa setup that is used to test the sera. Such an assay based on a polyclonal antibody as the capture antibody, makes it difficult to standardize the assay and bring it to market. What was the rationale for this approach? Why did the authors not use the Nb developed for this capture? Was this solely related to the fact that the Nb based assay could not detect NS1 in clinical samples? Since only one Nb was finally assessed for capture of NS1 in clinical samples, did the authors test other Nb from their initial panel if these could capture NS1 in clinical samples?

**Response:**

We are aware of the limitations of using polyclonal serum as a capture reagent. However, due to the inability of the selected Nbs to capture NS1 protein from patient samples, we chose to proceed with the development of the detection system using Nbs solely as detection antibodies.

In the future, we aim to select new Nbs—potentially by using NS1 protein derived from infected patient samples—that possess the ability to capture the antigen directly from clinical specimens.

The previously selected Nbs were not used as capture reagents because they lacked capture capability. All selected Nbs were tested for their ability to capture NS1 protein from clinical samples, but none demonstrated effective binding. The information has been added as S12 Fig.

The following text has been added:

In Methods:

**Lines 301-304**: *All Nbs were also evaluated as capture antibodies—both in their monovalent form and as Fc-fusion constructs—using clinical serum samples, following the establishment of the ELISA protocol (see Optimization of the Nanobody-based ELISA).*

In Results:

**Lines 558-561:** *Nanobodies from the initial panel, assessed in both their monovalent and Fc-fused formats, were evaluated for their ability to capture NS1 in clinical serum samples. Under the assay conditions employed, none of the candidates exhibited detectable NS1 capture (see S11 Fig).*

Figure legend:

**Lines 1319-1323:** *S11 Fig: Evaluation of monovalent and Fc-fused Nanobodies for NS1 detection in clinical serum samples. The ability of Nbs, in both monovalent and Fc-fused formats, to capture NS1 from clinical serum samples was assessed using the established cNb-ELISA protocol. None of the tested Nbs demonstrated detectable NS1 capture under the conditions employed.*

**Conclusions**

-Are the conclusions supported by the data presented?

-Are the limitations of analysis clearly described?

-Do the authors discuss how these data can be helpful to advance our understanding of the topic under study?

-Is public health relevance addressed?

**Reviewer #1:** The manuscript briefly mentions limitations (e.g., lower detection of DENV-4 NS1) but does not thoroughly explore their implications or potential solutions. The limitation regarding DENV-4 detection is acknowledged but not deeply analyzed or addressed.

**Response:**

We tried to better interpret our results and have added more information after revising available bibliography related to detection problems as follows:

**Lines 833-841:** *NS1 antigen sensitivity has been shown to vary across different epidemiological contexts. Felix et al. reported that secondary DENV-2 infections may have contributed to a high rate of false-negative results during the 2010 dengue outbreak in Brazil [105]. Sequencing of NS1-positive and -negative isolates revealed no mutations to explain the diagnostic failure. Koraka et al. later suggested that reduced NS1 detection in secondary infections may result from the sequestration of NS1 protein within immune complexes, limiting its availability for capture [106]. In our case, immune complex dissociation treatments were applied only to the pooled positive serum samples using Nbs as the capture element. We were unable to perform this treatment on individual samples in the cNb-ELISA due to the limited volume of sample available for testing.*

The writing could better position the study within the broader context of existing diagnostic approaches by comparing the results and methods with other current diagnostic tools. There is limited discussion on how the nanobody-based ELISA compares to other NS1 detection methods or commercial diagnostic kits.

**Response:**

We have already compared the performance of our cNb-ELISA with commercial kits (see lines **708–715**). However, we were unable to evaluate its performance against other diagnostic methods, such as PCR—commonly used in public hospitals—because the samples available to us had not been tested using these or other reference tools.

Additionally, we compared the cNb-ELISA with other nanobody-based detection systems and included the following text:

**Lines 714-715:** *A common limitation in prior studies is the use of recombinant proteins, spiked serum, or a limited number of infected human samples—insufficient for robust diagnostic validation.*

Some sentences are awkwardly constructed or contain minor grammatical errors that may detract from the manuscript's professionalism. For example, "Considering the restrictions of the public health system in accessing commercially available diagnostic kits" could be rephrased for clarity.

**Response:**

We thank the reviewer for this suggestion. Access to diagnostic kits in Argentina is severely limited, which may be difficult to fully grasp from an outside perspective. Only a few reference hospitals have the resources to perform these types of tests. For example, the Tropical Diseases Laboratory—which provided the sample set used in this study—no longer has access to such detection systems. The text has been rephrased for clarification as follows:

**Lines 47-48:** *Given the limited access to commercially available diagnostic kits within the public health system.*

**Reviewer #2:** Yes to all the questions

**Reviewer #3**: - A general statement is made about the cost of monoclonal antibodies compare to the cost of Nb. It would be good if this could be substantiated by actual numbers and how this would translate to the actual difference in cost per test. Especially given that the Nb also need to be modified with biotin for the test and that the capture antibody is a polyclonal Ab, and that for the detection antibody a Nanobody HRP fusion that is produced in eukaryotic cells is used. The latter two aspects seem to abolish the possible cheaper cost of using Nb.

**Response:**

Numerous studies suggest that Nb production is significantly more cost-effective than mAb production, although direct numerical comparisons are scarce. This variability likely reflects differences in reagent costs across countries. In our case, bacterial expression of Nbs is considerably more economical than using mammalian cell lines, due to the high cost of media and additives required for eukaryotic systems.

Although not detailed in this paper, we successfully produced anti-NS1 Nbs fused to alkaline phosphatase in *E. coli*. However, these constructs yielded low signal-to-noise ratios and were therefore not used to test valuable patient samples. Based on our experience, a more cost-effective strategy may involve producing HRP-conjugated Nbs in stable mammalian cell lines, which would at least reduce transfection-related expenses.

To support our claims regarding the cost advantages of Nbs over mAbs, we have included two recent review references in **line 707**.

Discovery of nanobodies: a comprehensive review of their applications and potential over the past five years. 10.1186/s12951-024-02900-y. Reference 69.

Cost and supply considerations for antibody therapeutics, 10.1080/19420862.2025.2451789. Reference 70.

- One aspect that does not seem to be considered is regulatory aspects / approval of diagnostic devices. Are there actual diagnostic devices on the market based on nanobodies? It would be good if this could be addressed as this would show the value and viability of the idea of making Nb based diagnostic assays for antigens.

**Response:**

We appreciate this insightful comment, which has helped us strengthen our manuscript. In response, we have incorporated the following text into the discussion section:

**Lines 848-855***: Rotadial represents the first Nb-based immunoassay approved for diagnostic use in Argentina, specifically developed to detect Group A Rotavirus in pediatric fecal samples. Utilizing a sandwich ELISA format with Nbs targeting the VP6 protein, the assay demonstrated 100% sensitivity and 99% specificity when benchmarked against commercial kits [107]. Rotadial has been incorporated into Argentina’s national diagnostic network, with thousands of units distributed to hospitals participating in the Red Nacional de Vigilancia de Gastroenteritis Virales [108]. Its implementation underscores the practical viability, affordability, and diagnostic robustness of Nb-based platforms in clinical settings.*

- The authors mention using stable HEK-293T cells to express Nanobody-HRP fusions. Why HEK-293T cells to do this, usually these cells are used only for transient expression?

**Response:**

We initially referred to HEK293T cells, as both our group and others have successfully used them for transient expression of nanobody-HRP fusions. However, it is true that CHO cells are more commonly employed for stable protein expression. Accordingly, we have revised the text to reflect this distinction:

**Lines 750-753:** *Additionally, transiently transfected HEK-293T cell lines have been successfully used to express Nb-HRP fusions [81,82]. Establishing a stable CHO cell line for this purpose could further reduce production costs and streamline the generation of Nb-based detection reagents.*

- Line 712: this part is quite speculative, and if statements are to be made about negative samples that might be considered positive, then probably other clinical samples need to be used that are better validated, or these samples need to be thoroughly tested using different assays to be sure if they are positive or negative. In this context, it could have been interesting to compare the results obtained with the ELISA-based kits with PCR experiments to determine if the specificity and sensitivity of the serological and molecular methods can be compared.

**Response:**

We fully agree with the reviewer on the importance of more robust validation of negative samples. However, the primary constraint in this study arises from the diagnostic algorithm implemented at the hospital where the samples were collected. Specifically, specimens obtained after day 5 of symptom onset were not processed using the standard diagnostic kit (Platelia) typically reserved for early-phase samples (days 0–5).

At present, the laboratory that supplied these samples no longer has access to the Platelia kit, rendering retrospective analysis unfeasible. Additionally, PCR data for this cohort is regrettably unavailable, further limiting our ability to perform confirmatory testing. To partially address this issue, we tested a subset of samples using the DIA.PRO kit. Notably, the results from our assay showed better correlation with the DIA.PRO kit than with the outcomes derived from the hospital’s algorithm, particularly for samples previously classified as negative.

- Building on the previous point, it could have been of interest to include in the patient cohort also the samples positive for Dengue IgM, to try to determine the window in which the assay can be used.

**Response:**

We sincerely appreciate the reviewer’s insightful question. Unfortunately, access to patient samples is highly restricted, which poses significant logistical challenges. In the specific case of the samples analyzed in this study, a direct comparison with IgM results is not feasible. This limitation stems from the diagnostic protocol followed at Orán Hospital, where NS1 antigen detection and IgM antibody testing are conducted at distinct time points in the disease progression.

As previously noted, NS1 detection is performed from symptom onset up to day 5, whereas IgM testing is carried out from day 5 through day 21. Consequently, the same sample is not subjected to both assays, and no overlap exists in the testing windows. This protocol inherently prevents simultaneous evaluation or extension of NS1 detection beyond its designated timeframe. To make matters even more complicated, due to government restructuring and budget cuts, the Tropical Diseases Laboratory is no longer part of the Provincial Commission on Health Science Research (COPICSA), and currently the staff is either no longer working in epidemiological surveillance or has been reassigned, making it extremely difficult to obtain new samples or data from old ones.

- For the future, urine and plasma samples could also be collected, as it appears that NS1 can be detected for a longer period in as it appears that NS1 can be detected for a longer period in these specimens. Maybe this can be discussed?

**Response:**

According to Andries et al. (10.1371/journal.pntd.0004100), NS1 protein concentrations in clinical specimens range from 0.8 ng/mL to 8 µg/mL in plasma, and from 0.5 ng/mL to 60 ng/mL in urine. The limit of detection (LOD) of our cNB-ELISA system varies between 101.8 and 507 ng/mL depending on the DENV serotype. Based on these values, our assay is suitable for detecting NS1 in plasma but is not sensitive enough for reliable detection in urine samples. Therefore, we have not explored the application of our kit for urine-based diagnostics. Future improvements in assay sensitivity may enable its use for such sample types.

Editorial and Data Presentation Modifications?

Use this section for editorial suggestions as well as relatively minor modifications of existing data that would enhance clarity. If the only modifications needed are minor and/or editorial, you may wish to recommend “Minor Revision” or “Accept”.

**Reviewer #1:** (No Response)

**Reviewer #2:** Before discussing the major issues, let me start with a few easy to amend suggestions:

Line 156 Is the recombinant NS1 the intact gene, does it contain a secretion signal?

**Response:**

The information about secretion signal is mentioned in the text:

**Lines 174-178:** *Two groups of constructs were used to clone NS1 from all four DENV serotypes: a pCAGGS-ss vector containing the human serum albumin secretion signal and a His-tag [42], and a modified pFUSE vector (invivoGene) with an IL2 secretion signal, a Flag-tag at the N-terminus, and a His-tag at the C-terminus. Recombinant NS1 protein from Zika virus (ZIKV, MR766) was also cloned into the same vectors.*

Line 171: please add the interval of the boosts and/or timing of boosts. The volume was 250 microL, but what was the concentration?

**Response:**

It was not possible to determine the concentration of NS1 in the sample that was injected. But to improve concentration the supernatant was concentrated 4 times.

This information has been added in:

**Line 162:** *four times.*

The information regarding interval of the boost was previously included in S1 fig, however we have added timing bust in Methods.

**Line 194:** *On day 28, 41, 58. 83, 90*.

Line 176 what is the source of the anti-lama IgG HRP conjugate? or how was it made? Writing that it was 1:15,000 diluted is not very helpful if we ignore the original source an/or activity.

**Response:**

We have used Goat anti-llama antibody, HRP-conjugated Bethyl Laboratories (cat. no. A160), dilution was done according to provider suggestion.

The following information was added:

**Line 199:** *(Bethyl Laboratories, 1:15.000)*

Line 186: You mention 'counter selection steps' but it is not clear what these are or how they were performed.

**Response:**

A detailed protocols for panning including information on counter selection steps were included:

**Lines 208-218:** *Welles prepared for both selection strategies were incubated at room temperature for 1 hour with 2% skimmed milk in PBST. Recombinant phages were then subjected to three successive rounds of counter-selection, each involving a 15-minute incubation at room temperature of 200 µL of phage suspension (at a concentration of 10¹² phages/mL) in wells containing protein extracts derived from non-transfected HEK293T or Vero cells, as appropriate. Counter selection steps were included to diminish non-specific phage binding, as the llama was immunized with a supernatant containing several irrelevant proteins. After the final counter-selection step, 100 µL of the collected phage suspension was transferred to the selection wells containing either the recombinant NS1-1 protein or transfected Vero cells. The remaining 100 µL was added to wells containing protein extracts from non-transfected HEK293T or Vero cells, serving as negative controls.*

Line 199: "Plates were blocked with PBST" normally BSA, casein, or protein free blocking agents are used. Only PBST? that is strange and would not be considered as blocking residual protein binding sites.

**Response:**

Thanks for pointing this out. The text has been now corrected to:

**Lines 234-235:** *The next day, plates were blocked with* *3% skimmed milk in PBST, then incubated with 100 µL of periplasmic extract for 2 hours at room temperature.*

Line 287: "at specific intervals" this is everything except "specific" or informative.

**Response:**

We appreciate this correction. The following text was modified:

**Lines 337-341:** *A rabbit was subjected to a five-dose immunization protocol over a four-month period. The first two immunizations (day 0 and day 28) used 200 μg of pCAGGS NS1-1, NS1-3, and NS1-4 plasmids. A protein booster (30 µg of recombinant NS1-1, NS1-3, and NS1-4) was administered at day 56. The final two immunizations (day 91 and day 119) used 200 µg of pCAGGS NS1-1 plasmid.*

Line 378: it is strange (not-standard) to use equine serum as blocking agent. At what dilution was it used? Presumably this contains ca 20-50 mg Serum albumin/ml. So was the final concentration of HSA around 1% or 3% (comparable to BSA?) then why doesn't it give the same result as BSA?

**Response:**

We thank the reviewer for raising this point. Equine serum was tested as an alternative blocking agent at a 5% (v/v) dilution as it is suggested by different providers such as thermoFisher: <https://www.thermofisher.com/antibody/product/Normal-Horse-Serum-Control/31874>, or Vector Labs: https://vectorlabs.com/products/normal-horse-serum-blocking-solution/.

Equine serum is a complex mixture of proteins, lipids, and other serum components, not purified albumin. We suspect that the presence of these additional components may interfere with antigen–antibody interactions or fail to adequately block nonspecific binding sites in our assay format. This is likely to explain why equine serum gave very low absorbance values compared to BSA or skim milk. For clarity, we have now specified the dilution used in the Methods section:

In Methods:

**Lines 292-293**: *Plates were blocked for 1 hour at room temperature with 5% equine serum, 3% BSA, 1% BSA, or 3% milk in PBST.*

In results:

**Lines 442-444:** *Optimal assay performance was determined by comparing two microplate brands and three blocking reagents (3% and 1% BSA, 5% equine serum, and 3% skimmed milk).*

Fig 1A was also modified to include this information.

Major comments:

Thus, it seems that the Nb as NS1 capturing agent is not working efficiently with clinical serum samples, possibly due to the presence of host IgGs of the infected patient. Therefore, the capturing Nb was substituted with polyclonal rabbit anti-NS1 serum. However, this is not a sustainable source of antibodies. So, how well can this polyclonal reagent be upscaled to be used in the clinic over a longer period, and will a new batch from another rabbit provide reproducible and comparable results?

**Response:**

Our goal was to develop an ELISA assay based exclusively on Nbs. We successfully demonstrated that the selected Nbs function effectively for both capture and detection of the recombinant protein produced in eukaryotic cells. Surprisingly, the Nbs failed to detect the protein in samples from infected patients—although they did work in serum samples artificially spiked with the recombinant protein. This discrepancy highlighted a key limitation in our current approach. We are aware of the constraints associated with using polyclonal sera for capture. However, at this stage, it was the only method that allowed us to show that the selected Nbs can be applied in various formats for detecting infected samples. As discussed, we have now revised our Nb selection strategy. Specifically, we are implementing a panning protocol based on samples from infected patients to improve capture performance. We hope this will enable us to identify Nbs that are truly capable of detecting patient-derived samples in future assays.

The discussion is much too long and repetitive: for example, the widespread and increasing frequency of occurrence of Dengue infections has been mentioned in the Introduction section. There is no need to repeat it in the discussion.

**Response:**

The text has been revised to remove redundancy and improve clarity.

Also, the results were clear enough, so that there is no need to repeat the set-ups that didn't work (plastic binder peptides).

**Response:**

Information about plastic binding peptides has been removed.

The authors mention that the commercialised diagnostic Dengue kits are expensive and not that easy to obtain in South America. Therefore, this in-house system is claimed to be easier accessible and more economic for the clinical labs. Now, clincal labs are not keen to change the reagents (or provider of their reagents) as it would mean that they should repeat all or most of their previous samples, to guarantee that the same results will be obtained. Therefore, if you want to convince clinical labs to change you absolutely need to demonstrate the sensitivity and specificity of your protocol or kit with that of the commercial widespread kits using exactly the same well documented clinical samples. Without this information nobody will ever try this new set of reagents. (and also they need to be ensured that the reagents will remain available over longer periods (decades).

**Response:**

We are fully aware of the limitations of our current detection system. Unfortunately, obtaining patient samples in sufficient quantities to repeat validation procedures is extremely challenging. Public hospitals—where most diagnostic testing is conducted—typically lack freezer storage facilities, meaning samples are often discarded shortly after collection. Due to these constraints, we had to rely on the sample bank at the Tropical Diseases Laboratory in Salta. However, even at this institution, the volume of samples available for research is very limited. At this stage, we do not intend to commercialize the detection system in its current form. We still need to select new Nbs capable of capturing the NS1 protein in samples from infected patients. Our primary goal is to demonstrate the capacity to develop diagnostic reagents within our country, using materials produced in our own laboratories. In the future, we aim to commercialize these locally developed tools.

Reviewer #3: (No Response)

**Summary and General Comments**

Use this section to provide overall comments, discuss strengths/weaknesses of the study, novelty, significance, general execution and scholarship. You may also include additional comments for the author, including concerns about dual publication, research ethics, or publication ethics. If requesting major revision, please articulate the new experiments that are needed.

**Reviewer #1**: (No Response)

**Reviewer #2:** A new type of ELISA was developed to screen clinical samples on the presence of Dengue NS1. The authors developed a polyclonal rabbit anti-NS1 IgG as capturing agent and used an anti-NS1 Nb conjugated to HRP as detecting agent.

A few details in the methodology are missing and should be added. The discussion should be shortened.

**Response:**

New protocols have been added to the methodology, and missing information has also been incorporated into the existing sections. The discussion section has been shortened in accordance with the reviewers’ request.

The experimental work was well explained, and the authors assessed the performance of a number of Nb modifications (plastic binder peptides, IgG reconstruction, biotinylation).

Apparently only the use of rabbit polyclonal anti-NS1 as capturing agent is working with samples from putative infected patients. This might affect the sustainability of the protocol, if it will be used on a larger scale. So the authors are invited to discuss, how they would be able to cope with this issue.

**Response:**

As previously mentioned, we acknowledge that a major limitation of this development is the need to use polyclonal serum for antigen capture. We propose conducting a new selection of nanobodies using patient-derived samples, as NS1 protein in these specimens is likely to be in its native conformation and physiological context, making it more suitable for detection. It is possible that the protein used during the various rounds of panning—whether recombinant purified protein or protein expressed in transfected cells—does not fully replicate the characteristics of NS1 found in patient samples, which may have negatively impacted detection performance. We have discussed this in the Discussion section:

**Lines 769-777:** *A more effective approach might involve using a selection strategy directly with sera from positive patients, while counter-selecting with negative sera, rather than relying on recombinant protein. We are currently adopting this new strategy, as it seems promising for selecting Nbs with strong capture properties from the existing library. This is because the llama was immunized with protein purified from the supernatant of C6/36 insect cells, which are known to produce the soluble hexameric form of NS1 [77]. The primary motivation for this approach is that developing a detection system for DENV-infected patients relying solely on Nbs would significantly lower production costs and enhance the reproducibility of detection, a known challenge when using polyclonal sera.*

**Reviewer #3:** - The paper is overall well written. The experimental setup is straightforward, and the authors provide detailed explanations for the reasons behind the experiments described in the paper.

- The approach, based on nanobodies instead of monoclonal antibodies, is innovative and has the potential to find a place on the market.

- Several aspects related to the methods used and the results are not sufficiently clear and need further clarification:

**Response:**

New protocols have been added to the methodology, and missing information has also been incorporated into the existing sections.

- Conclusions and some statements are not always convincingly supported by own data and/or existing literature.

**Response:**

Additional references have been incorporated, and the discussion section has been further refined to enhance clarity and depth.

PLOS authors have the option to publish the peer review history of their article (what does this mean?). If published, this will include your full peer review and any attached files.

If you choose “no”, your identity will remain anonymous but your review may still be made public.

Do you want your identity to be public for this peer review? For information about this choice, including consent withdrawal, please see our Privacy Policy.

Reviewer #1: No

Reviewer #2: No

Reviewer #3: No

[NOTE: If reviewer comments were submitted as an attachment file, they will be attached to this email and accessible via the submission site. Please log into your account, locate the manuscript record, and check for the action link "View Attachments". If this link does not appear, there are no attachment files.]

Figure resubmission:

While revising your submission, please upload your figure files to the Preflight Analysis and Conversion Engine (PACE) digital diagnostic tool, https://pacev2.apexcovantage.com/. PACE helps ensure that figures meet PLOS requirements. To use PACE, you must first register as a user. Registration is free. Then, login and navigate to the UPLOAD tab, where you will find detailed instructions on how to use the tool. If you encounter any issues or have any questions when using PACE, please email PLOS at figures@plos.org. Please note that Supporting Information files do not need this step. If there are other versions of figure files still present in your submission file inventory at resubmission, please replace them with the PACE-processed versions.

**Response:**

The figures have been thoroughly reviewed and successfully passed the quality control criteria.

Reproducibility:

To enhance the reproducibility of your results, we recommend that authors of applicable studies deposit laboratory protocols in protocols.io, where a protocol can be assigned its own identifier (DOI) such that it can be cited independently in the future. Additionally, PLOS ONE offers an option to publish peer-reviewed clinical study protocols. Read more information on sharing protocols at https://plos.org/protocols?utm_medium=editorial-email&utm_source=authorletters&utm_campaign=protocols
